# Active plasma renin concentration throughout healthy and complicated pregnancy: a systematic review and meta-analysis

**DOI:** 10.1186/s12958-024-01200-2

**Published:** 2024-03-07

**Authors:** Wisal El Fathi, Maaike van Ochten, Munieb Rehman, Sander M. J. van Kuijk, Joanna IntHout, Chahinda Ghossein-Doha, Sander de Haas, Marc E. A. Spaanderman, Joris van Drongelen

**Affiliations:** 1 Department of Gynecology and Obstetrics, Radboud University Medical Center, Nijmegen, The Netherlands; 2 Department of Cardiology, Isala Hospital, Zwolle, The Netherlands; 3 Department of Clinical Epidemiology and Medical Technology Assessment, Maastricht University Medical Center, Maastricht, The Netherlands; 4 Department for Health Evidence, Section Biostatistics, Radboud University Medical Center, Nijmegen, Netherlands; 5 Department of Cardiology, Maastricht University Medical Center, Maastricht, The Netherlands; 6 Department of Gynecology and Obstetrics, Maastricht University Medical Center, Maastricht, The Netherlands

**Keywords:** Renin, Renin-angiotensin system, Pregnancy, Complicated pregnancy, Hypertension

## Abstract

**Background:**

Pregnancy is characterized by profound circulatory changes and compensatory adjustments in the renin-angiotensin-aldosterone system (RAAS). Differences in regulatory response may antedate or accompany vascular complicated pregnancy. We performed a systematic review and meta-analysis to delineate the trajectory of active plasma renin concentration (APRC) in healthy pregnancy and compare this to complicated pregnancy.

**Methods:**

We performed a systematic review and meta-analysis on APRC during normotensive and hypertensive pregnancies, using PubMed (NCBI) and Embase (Ovid) databases. We included only studies reporting measurements during pregnancy together with a nonpregnant reference group measurement. Risk of bias was assessed with QUIPS. Ratio of the mean (ROM) and 95% confidence intervals (CI) of APRC values between pregnant and nonpregnant women were estimated for predefined intervals of gestational age using a random-effects model. Meta-regression was used to analyze APRC over time.

**Results:**

In total, we included 18 studies. As compared to nonpregnant, APRC significantly increased as early as the first weeks of healthy pregnancy and stayed increased throughout the whole pregnancy (ROM 2.77; 95% CI 2.26–3.39). APRC in hypertensive complicated pregnancy was not significantly different from nonpregnancy (ROM 1.32; 95% CI 0.97–1.80).

**Conclusion:**

Healthy pregnancy is accompanied by a profound rise in APRC in the first trimester that is maintained until term. In hypertensive complicated pregnancy, this increase in APRC is not observed.

**Supplementary Information:**

The online version contains supplementary material available at 10.1186/s12958-024-01200-2.

## Background

The renin-angiotensin-aldosterone system (RAAS) plays an essential role in the regulation of blood pressure and fluid- and electrolyte balance. The first hormone in this cascade, renin, is predominantly released by the juxtaglomerular cells of the kidneys in response to a reduction in arterial pressure and/or the sodium load in the distal tubule (a reflection of reduced effective circulating volume) [1]. Renin converts angiotensinogen into angiotensin I, which is subsequently converted into angiotensin II (ANGII). This hormone induces systemic arterial vasoconstriction and stimulates the release of aldosterone from the adrenal cortex. Aldosterone makes the renal tubules reabsorb sodium and with it, water into the blood vessels. Through this pathway, the RAAS controls blood volume and arterial blood pressure and is of major importance in the regulation of hemodynamic changes [1].

Healthy pregnancy is characterized by an significant drop in peripheral resistance leading to such hemodynamic changes. To maintain a stable blood pressure, the maternal body increases the cardiac output by augmentation of the heart rate and stroke volume, and enhances fluid retention through activation of the RAAS [2]. Inadequate adaptation of these circulatory responses predisposes to gestational hypertensive complications [2–4]. As the RAAS is a major key modulator in hemodynamic regulation, understanding the circulatory changes during pregnancy and the contribution of RAAS is essential.

The change in renin concentration is likely to be of major importance during pregnancy, as it is the first step in RAAS activation. Several studies report an increase of the renin concentration during pregnancy from week five of gestation until term [5–7]. However, the course of the renin concentration throughout pregnancy is not known. Additionally, it is unclear to what extent an abnormal course of renin production is related to vascular maladaptation and thus gestational hypertensive disease. Profound insight in the course of renin during pregnancy may be pivotal to understand the underlying physiology in adaptive and maladaptive pregnancy.

To this end, we performed a systematic review and meta-analysis to describe the physiological time course of active plasma renin concentration (APRC) during healthy pregnancy. In addition, we investigated the time course of APRC in hypertensive complicated pregnancy.

## Methods

Our study followed the PRISMA guidelines for systematic reviews [8]. The study protocol was registered in the International prospective register of systematic reviews (registration ID: CRD42023442691) [9].

### Literature search

We performed a systematic literature search to collect published data on APRC during healthy and complicated pregnancies. The search was conducted in PubMed and Embase to find relevant literature from inception to March 2023, using the following keywords: ‘pregnancy’, ‘pregnancy induced hypertension (PIH)’, ‘pre-eclampsia (PE)’, ‘HELLP syndrome’, ‘gestational diabetes’, ‘fetal growth restriction (FGR)’, ‘small for gestational age (SGA)’, ‘RAAS’ and ‘renin’. There was no restriction based on publication date. The full search strategy is depicted in Appendix S1. The reference lists of reviews and included studies were searched to identify additional studies.

### Study selection

Two authors (W.E.F. and M.R.) screened the articles individually and independently based on the title and/or abstract and subsequently on full text. Any discrepancies were resolved by mutual consensus. Studies were included if they reported APRC as mean with standard deviation (SD), standard error (SE), 95% confidence interval (CI) or median and interquartile range (IQR) during healthy and/or complicated pregnancies. Studies were only included if they also measured the APRC in a reference group (≥ 6 weeks postpartum, before conception or in nonpregnant controls). The last postpartum measurement was used if studies reported more than one reference measurement postpartum. Written in another language than English or Dutch, being a case report or a review, and having no full-text available were reasons for exclusion. In addition, articles only reporting on measurements from patients with pre-existing diabetes or cardiovascular disease were excluded. Studies were excluded if subjects used medication or were subjected to an intervention that could influence APRC at the time of the study. Iron and vitamins were considered not to influence APRC.

### Data extraction

Data extraction was performed by two authors (W.E.F. and M.O.). We extracted the following characteristics from the included studies: study design, sample size and the method and circumstances of measuring APRC. Furthermore, information on age, weight, height, blood pressure, gravidity, parity, duration of pregnancy and APRC as mean or median (with SD, SE, 95% CI or IQR) was extracted from the pregnant and nonpregnant subjects. Data presented in graphs were extracted with the use of software [10].

### Quality assessment

A quality assessment was performed by two independent reviewers (W.E.F and M.R.) with a self-adjusted version of the Quality In Prognosis Studies (QUIPS) tool to score the articles based on different domains (study participation, study attrition, variable measurement, data reporting, and study design) [11], as can be seen in Table 1. A score of > 60% was defined as high quality, 30–60% as moderate quality and < 30% as low quality.

**Table 1:** Modified QUIPS tool for assessment of study quality. The individual studies are defined as a high quality study (HQ), moderate quality study (MQ), or low quality study (LQ)

Domain	Items for consideration	Al Kadi (2005) [20]	Baker (1992) [21]	Brown (1990) [24]	Brown (1992a) [25]	Brown (1992b) [27]	Brown (1993) [26]	Brown (1994) [22]	Brown (1995) [23]	Derkx (1987) [28]	Jarvis (2012) [29]	Langer (1998) [30]	Lewandowski (2023) [35]	Nicholson (1987) [5]	Pedersen (1982) [31]	Skinner (1972) [32]	Spaan (2013) [33]	Spaanderman (2001) [7]	Thomsen (1993) [34]
**Study Participation**	*Adequate description of participant characteristics:*																		
	Parity or gravidity	+	–	+	–	+	+	–	–	–	–	+	–	+	–	–	+	+	+
	Health or comorbidities of participants	+	+	–	–	+	–	–	+	–	+	+	–	+	–	+	+	+	+
	Clear reporting of weeks amenorrhea	+	+	+	+	+	+	–	+	+	+	+	+	–	–	+	+	+	+
	Ethnicity	–	+	–	–	–	–	–	–	–	–	–	–	–	–	+	–	+	–
	Height	–	–	+	–	+	+	–	+	–	+	–	–	–	–	–	–	+	–
	Non-pregnant weight or BMI	+	–	–	–	–	–	–	–	–	+	–	–	–	–	–	–	+	–
	Use of medication or supplements	+	+	+	+	+	+	+	+	+	+	–	–	+	+	+	+	+	+
	Adequate description of participant recruitment	+	–	+	+	+	–	+	+	–	–	+	–	–	+	–	+	+	+
	Adequate description of inclusion and exclusion criteria	+	–	–	–	–	–	–	+	–	+	+	+	–	+	–	+	+	–
**Study Attrition**	Reasons for loss to follow-up/drop-out are provided	?	?	?	?	?	?	?	?	–	?	?	?	?	?	?	–	+	–
	Adequate description of participants lost to follow-up / differences between participants who completed and drop-outs	?	?	?	?	?	?	?	?	–	?	?	?	?	?	?	–	+	–
**Variable Measurements**	Method of APRC measurement is valid and reliable	+	+	+	+	+	+	+	+	+	?	+	+	+	+	+	?	+	+
	The methods and setting are the same for all study participants and throughout follow up	+	+	+	+	+	+	+	–	+	+	+	+	?	+	+	+	+	+
**Data Reporting**	Time frame of measurements (gestational age) are reported as mean	–	–	+	+	+	+	–	+	–	+	+	+	–	–	–	+	–	–
**Study Design**	Study used a longitudinal study design	+	+	–	–	–	–	–	–	+	+	+	–	–	+	–	+	+	+
	Multiple (≥2) longitudinal measurements during pregnancy of APRC	+	+	–	–	–	–	–	–	–	–	+	–	–	–	–	–	–	+
	Baseline value was a prepregnant measurement of the variable	+	–	–	–	–	–	–	–	–	+	–	–	–	–	–	–	+	–
	Score (%)	71	47	47	35	53	41	24	47	29	59	59	29	24	35	35	53	88	53
	Quality	HQ	MQ	MQ	MQ	MQ	MQ	LQ	MQ	LQ	MQ	MQ	LQ	LQ	MQ	MQ	MQ	HQ	MQ

### Data analysis

Data on plasma renin measurements during a healthy or complicated pregnancy were categorized into five different intervals for gestational age (5–14, 15–21, 22–28, 29–35 and 36–41 weeks). We only included subgroups of more than 4 subjects. Data were documented as mean and SD. Values for APRC that were presented as 95% CI, SE or IQR were converted to SD. If the calculated skewness was < 0.5, medians and interquartile ranges were converted to means and SD using the method of Cochrane [12]; otherwise, the method of Hozo et al. was used [13].

The ratios of the means (ROMs) with the corresponding 95% CI of the APRC values were estimated to assess the association between gestational age and APRC during pregnancy as compared to non-pregnant values. The analysis was performed with a random effects model, with the restricted maximum likelihood (REML) estimator for the between-study variance tau and the Hartung-Knapp [14] adjustment to account for the limited number of studies. A clustering effect was added to account for the use of repeated measures at different gestational ages within studies. Some studies reported multiple results when they measured APRC with different methods or in different positions. In the main analysis, we only included the results corresponding to the setting and position that were most used in other studies. Sensitivity analyses were performed to evaluate the effect of the method of renin measurement, posture during sampling, moment of sampling, outcome measure (mean or median), study quality and type of reference group. Heterogeneity between the studies was evaluated using the I^2^ statistic. I^2^ values of less than 25%, between 25 and 50% and more than 50% were considered as low, moderate or high heterogeneity respectively [15]. We evaluated potential presence of publication bias with a funnel plot in combination with the Egger regression test [16].

A meta-regression analysis was performed to assess the course of the ROMs of APRC over time during pregnancy as compared to nonpregnancy. We used a mixed effects model with a linear time trend, a REML estimator for tau, and study as clustering effect to account for the use of longitudinal repeated measures within studies.

The statistical analyses were carried out with the statistical software R (version 4.1.3) [17] using the meta package [18] for the meta-analyses and the mixmeta package [19] for the meta-regression analysis with the clustering effect.

## Results

### Study and data selection

**Fig. 1:**
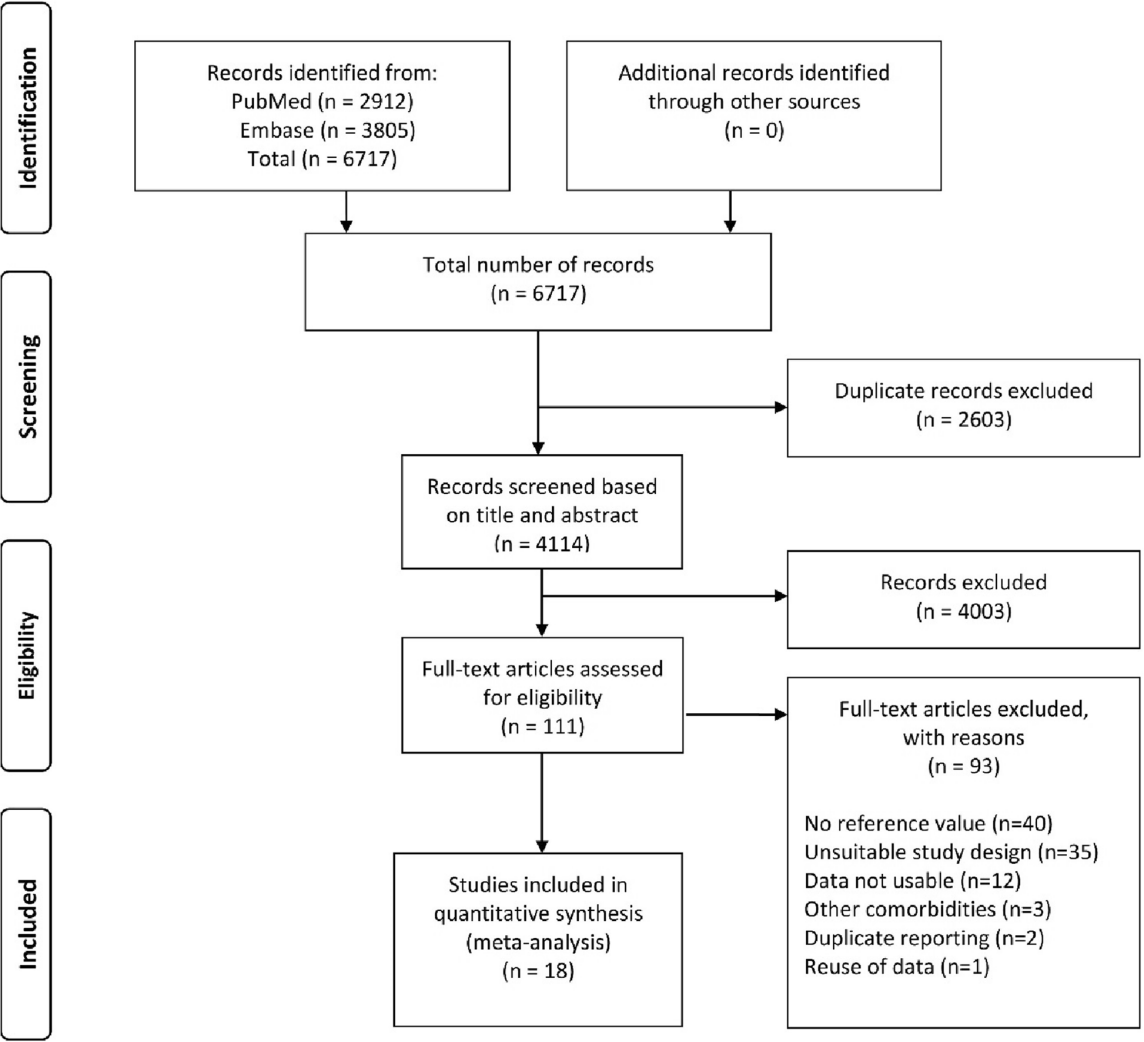
PRISMA flowchart of the study selection

Our search resulted in 6717 articles before removing all duplicates, and 4114 articles after removal of duplicates, see Fig. 1. After title and abstract screening, 111 remaining articles were assessed for eligibility. Studies were excluded if they did not report nonpregnant APRC reference values (*n* = 40), had an unsuitable study design (*n* = 35), presented unusable data (*n* = 12), reported comorbidities (*n* = 3), were a duplicate (*n* = 2), or reused already published data (*n* = 1).

In total, 18 articles met the inclusion criteria and were included for final analysis [5, 7, 20–35]. The included studies reported APRC measurements mostly in women during a healthy and/or complicated pregnancy due to PIH and PE. Two studies also mentioned SGA in babies of women with a healthy [33] and preeclampsia pregnancy [30]. Unfortunately, we were unable to identify articles that reported APRC measurements in pregnancies with gestational diabetes or FGR. Our search strategy did not identify articles that measured APRC during the first and second trimester of complicated pregnancies. Hence, we only found studies reporting APRC measurements during 29–41 weeks of pregnancy in patients with PIH and PE.

### Study characteristics

A general description of the study characteristics for both healthy and complicated pregnancies is given in Table S2.1 and S2.2, respectively. The study of Pedersen et al. did not include information about the gestational age and parity. However, we were able to reconstruct this from another study of this group that used the same subjects [36]. One study did not provide the gestational age of the subjects [22], but mentioned that the pregnant women were in third trimester of pregnancy. Another study measured APRC between 30 and 36 weeks of gestation and had, therefore, two overlapping intervals [5]. We categorized the APRC levels of these studies in the interval 29–35 weeks of gestation. Reference values for APRC were either nonpregnant control values (*n* = 9) or APRC measured prior to the pregnancy (*n* = 3) or postpartum (*n* = 6). The funnel plot (Fig. S3) and Egger’s regression test were not indicative of publication bias.

### Method and conditions of active renin measurement

The methods and circumstances of APRC measurements of the included studies are depicted in Table S2.3. Most studies measured APRC with an activity assay (*n* = 13), four studies used an immunoassay. One article reported that direct renin was measured. Therefore, we assumed that the immunoassay was used [29]. One study reported APRC measurements with both methods. For this study, we only included the APRC measured with an activity assay in the main analysis. The APRC measured with the immunoassay was included in the sensitivity analysis on type of renin assay. One article did not report the method of APRC measurement.

Most studies collected blood samples from participants in the lateral recumbency posture (*n* = 7). Four studies collected blood samples in the supine position, out of which two studies also collected blood samples when study subjects were tilted to 60 degrees upright or completely upright. For these studies, we included the APRC sampled in supine position in the main analysis and the upright samples in the sensitivity analyses for posture. In two other studies, participants were seated when blood was drawn. One article described that blood was drawn with the participants laid down, tilted a little to the left. The decubitus and semi prone position were reported only once, in two different articles. Lastly, two studies did not report the subjects’ posture at blood sampling.

Blood samples were mostly drawn in the morning (*n* = 12). One study collected blood samples around the middle of the day. Some studies reported that participants had been fasting before blood was collected for APRC measurement. Moreover, three studies reported that the subjects were on a constant sodium diet. However, most studies did not report whether the subjects had been fasting or using a diet before blood sampling.

### APRC during healthy pregnancy

**Fig. 2:**
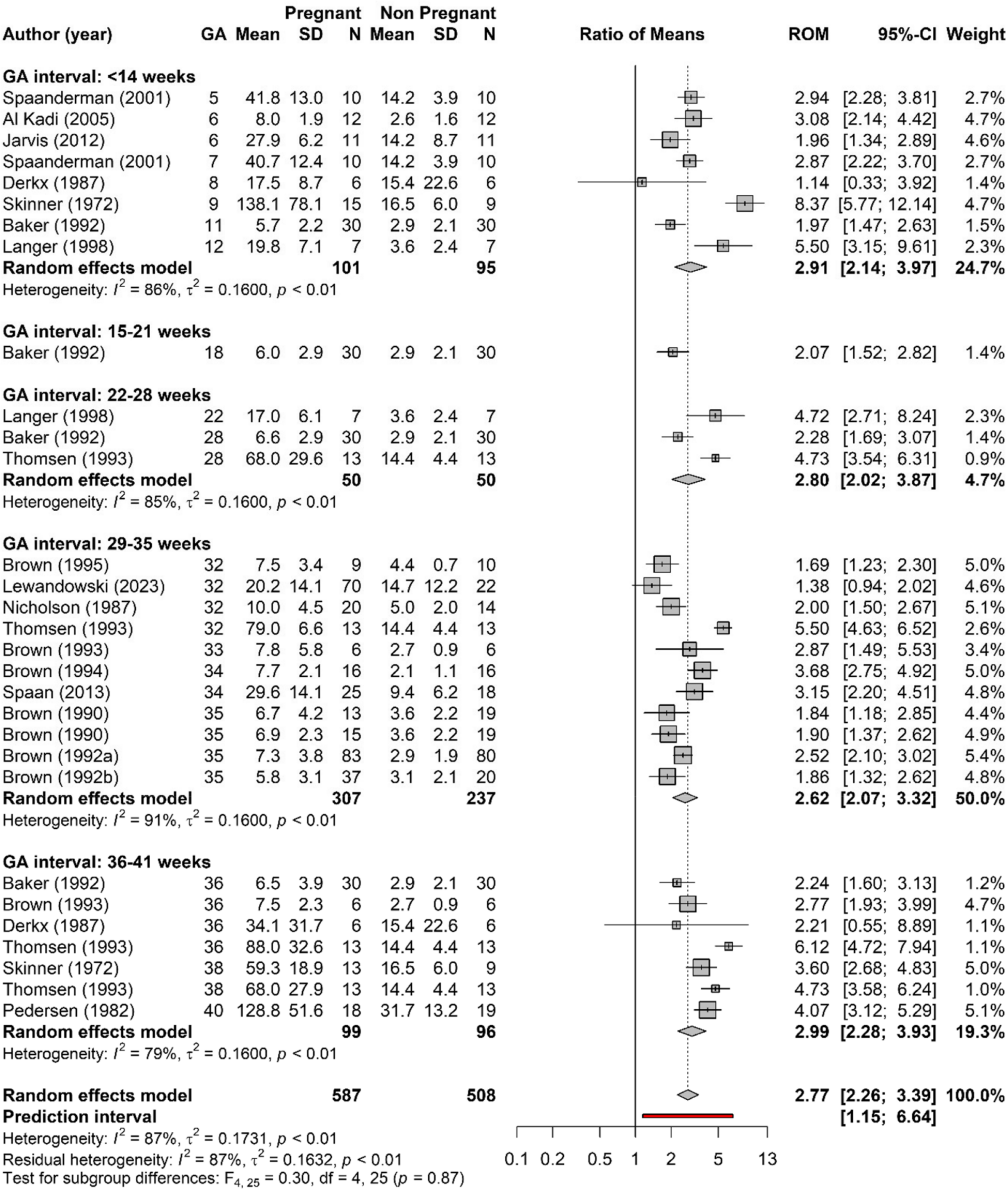
Forest plot of the ratio of means (ROM) of the active plasma renin concentrations (APRC) during healthy pregnancy at < 14 weeks, 15–21 weeks, 22–28 weeks, 29–35 weeks and 36–41 weeks of gestation compared to reference values in non-pregnancy, preconception or postpartum. Only studies with more than 4 subjects are included. Studies that are reported more than once provide data for different gestational weeks within the same study. Only the first author of each study is given. GA = gestational age in weeks, SD = standard deviation, CI = confidence interval

Figure 2 shows the forest plot of the ROM of APRC between the healthy pregnancy and the nonpregnant reference group. The meta-analysis shows a significant overall increase in APRC during pregnancy by a factor 2.77 (95% CI 2.26–3.39). This increase was similar and significant in all the different intervals of gestational age. There was only one study with more than 4 participants that measured APRC at interval 15–21 weeks of healthy pregnancy. High heterogeneity was observed between studies that measured APRC during healthy pregnancy.

We performed multiple sensitivity analyses on APRC during pregnancy (Appendix S4). We observed a smaller increase in APRC when we restricted the analysis to studies that measured renin with an immunoassay (ROM 2.18; 95% CI 1.32–3.58) (Fig. S4.3). Furthermore, we noticed that in the sensitivity analyses the increase in APRC was not always significant per interval as compared to the nonpregnant reference, but the overall results are comparable to those of the main analysis. Heterogeneity was still high in these analyses.

### APRC during complicated pregnancy

**Fig. 3:**
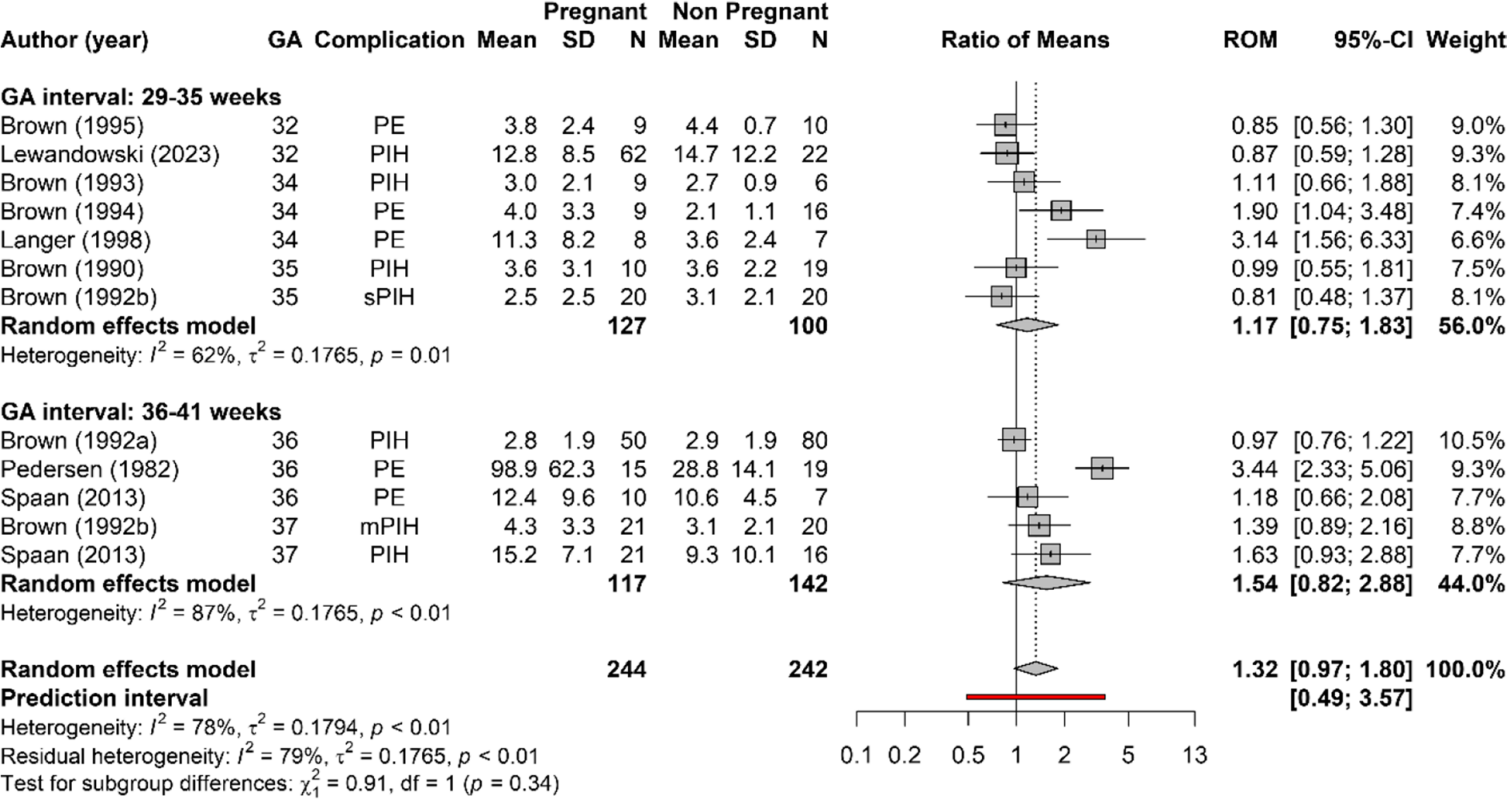
Forest plot of the ratio of means (ROM) of the active plasma renin concentrations (APRC) during hypertensive complicated pregnancy at 29–35 weeks and 36–41 weeks of gestation compared to reference values in non-pregnancy, preconception or postpartum. Studies that are reported more than once provide data for different gestational weeks within the same study. Only the first author of each study is given. GA = gestational age in weeks, SD = standard deviation, CI = confidence interval, PE = preeclampsia, PIH = pregnancy induced hypertension

Figure 3 shows the ROM in APRC between the pregnancies complicated by PIH and PE and the nonpregnant reference group. There was no significant difference in APRC between complicated pregnancy and nonpregnant participants (ROM 1.32; 95% CI 0.97–1.80). There was a high level of heterogeneity between studies that measured APRC during complicated pregnancies.

Sensitivity analyses were performed based on the reported complication (PE or PIH), method of renin measurement, posture during blood sampling and study quality (Appendix S5). There were not enough data to perform a subgroup analysis on data that were presented in median and interquartile range and data that were derived from graphs or only the studies that reported outcome as mean and SD. APRC in pregnancies complicated with PE seemed to be slightly more increased than in PIH complicated pregnancies (ROM 1.80 (95% CI 0.83–3.92) versus 1.07 (95% CI 0.85–1.34)) (Fig. S5.1). However, this increase was not significant compared to the nonpregnant reference group. Heterogeneity was still high in the sensitivity analyses.

### Meta-regression analysis

**Fig. 4:**
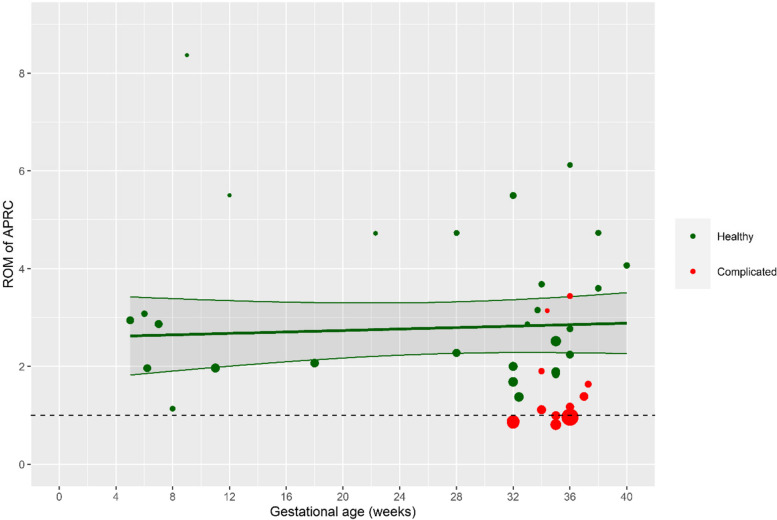
The ratio of means (ROM) of active plasma renin concentration (APRC) during pregnancy compared to nonpregnant women by gestational age. Shaded area represents the 95% confidence interval. The green data points represent healthy pregnancy; the red data points represent complicated pregnancy. Nonpregnant values were standardized as 1 and are represented by the dashed line

Figure 4 shows the results of the mixed-effects regression model. The ROM for APRC during healthy pregnancy is presented in green. Already in week 5 of pregnancy, mean renin concentration almost tripled compared to the nonpregnant APRC. The APRC stayed at this level until the end of the pregnancy. APRC in complicated pregnancy is shown in red and was noticeable lower than in healthy pregnancy.

### Quality assessment

Table 1 shows the quality assessment of the included studies. Two studies were defined as high, 12 studies as moderate and the remaining four articles as low quality. Most studies did not report a prepregnant APRC measurement (*n* = 15), nonpregnant weight or BMI (*n* = 15) or ethnicity (*n* = 15). Other frequently missing items were multiple longitudinal pregnant measurements (*n* = 14), information about height (*n* = 12) and an adequate description of inclusion and exclusion criteria (*n* = 10). Nevertheless, the majority of the studies did provide information about the use of medication or supplements (*n* = 16), the method of renin measurement (*n* = 16), setting (*n* = 16) and weeks of amenorrhea (*n* = 15).

## Discussion

Healthy pregnancy is accompanied by profound changes in central hemodynamic functions balanced by volume regulatory compensatory responses. To assess the role of changes in RAAS activation, we performed a systematic literature review and meta-analysis on APRC in pregnancy. We included 18 studies and observed that mean APRC almost triples during healthy pregnancy compared to non-pregnant APRC, which can be interpreted as a pivotal initiating renal hormonal response to counterbalance the immense drop in peripheral resistance. Furthermore, we found no significant increase in mean APRC in pregnancies complicated by PE or PIH, which indicates that these women have lower circulating levels of APRC during pregnancy compared to healthy pregnancy.

In almost all studies that measured APRC during healthy pregnancy, a significant increase in APRC was observed independent of gestational age. This increase can be caused by elevated levels of other hormones (e.g. relaxin, estrogen and progesterone), which are released by the ovaries, placenta and decidua, and contribute directly and indirectly to renin release [37]. At the same time, renin is synthesized by the juxtaglomerular cells of the kidneys in response to a subtle drop in afferent blood pressure, decreased glomerular filtration rate and lower circulating sodium chloride [38]. It remains to be determined which pathways contribute to the increase of APRC during pregnancy and to what extent.

Pregnancies that are complicated by PIH and PE are characterized by vascular maladaptation. In our study, we did not find a significant increase in APRC in complicated pregnancies as compared to nonpregnancy. Previous research found agonistic autoantibodies (AT_1_-AA) to the ANGII type 1 receptor (AT_1_R) in women with PE [39]. These AT_1_-AA suppress the release of renin, which may be an explanation for decreased APRC levels in vascular complicated pregnancies. Furthermore, AT_1_-AA interact with AT_1_R, which leads to vasoconstriction and therefore possibly hypertension [40]. Moreover, pregnancies complicated by PIH or PE show a significantly lower plasma volume expansion, probably associated with a lower RAAS activation [3]. Also, the high blood pressure observed in these complicated pregnancies might suppress the RAAS. However, it remains unclear if these lower levels of APRC precede PIH or PE, as we did not find studies that present APRC levels at an early gestational age. We recommend further research to focus on this part of pregnancy.

### Strengths and limitations

Our study describes the first meta-analysis on the effects of pregnancy on APRC. We used a systematic approach, in which we clearly defined the inclusion and exclusion criteria. All articles were screened and analyzed by two reviewers. Furthermore, we performed an extensive meta-analysis, including multiple sensitivity analyses to evaluate the influence of other factors. The sensitivity analyses revealed no significant influence of these factors on the outcome of our study, which contributes to the robustness of our results.

Besides these strengths, there are some possible limitations that need to be discussed. First, due to the different techniques used for renin measurements in the included studies, we needed to evaluated the increase in APRC as the ratio of mean values instead of absolute values. This may make interpretation of the results more difficult. The most used method was the activity assay, where renin is determined as the maximal velocity and production of angiotensin I [41, 42]. The immunoassay was the second most used method, where the APRC is determined with the help of antibodies that bind to renin [42]. For both methods, a converting factor is necessary to determine the exact APRC [42]. Unfortunately, most studies did not provide this factor and we therefore reported our results in relative terms. Second, APRC is influenced by several physiological conditions, such as posture during blood sampling or sodium intake. Our sensitivity analyses did not show an effect of posture on the observed outcome. Unfortunately, we were not able to investigate if diet has affected APRC as most studies did not describe whether the subjects were on a specific sodium diet. Third, the reference groups contained either nonpregnant control values or APRC measured prior to the pregnancy or postpartum. It is possible that APRC levels postpartum have not completely returned to the nonpregnant value, and we therefore may have underestimated the increase. Additionally, for the complicated pregnancy group one might argue that levels of healthy nonpregnant individuals or postpartum levels of non-healthy individuals could differ. However, our sensitivity analysis on the ROM of APRC showed no significant differences between these subgroups for both healthy and complicated pregnancies. Fourth, some of the included data were originally presented in graphs. In these cases, we extracted the values with a digital tool. This may have caused the results to be less precise, although exclusion of these studies did not change the estimated effect. Fifth, there was a considerable level of heterogeneity between studies. We aimed to decrease heterogeneity by applying strict inclusion and exclusion criteria, and performed multiple sensitivity analyses. However, we were not able to evaluate the effect of other factors such as sodium diet or patient characteristics, as this data was not available in the included literature. Furthermore, most studies were classified as low or moderate quality, which may have also contributed to the level of heterogeneity. Nevertheless, despite the heterogeneity between the studies, the majority of the studies showed an increase in APRC values during pregnancy.

## Conclusion

In summary, this is the first systematic review and meta-analysis that describes the course of APRC during healthy pregnancies and pregnancies complicated by PIH or PE. In healthy pregnancy, we observed a significant increase in APRC from week five of gestation until the end of pregnancy. This is in contrast to complicated pregnancies, where this increase was not observed. This suggests that healthy pregnancy is accompanied with an increase in APRC and that hypertensive complicated pregnancies are characterized by lower levels of APRC. The information provided by this study can be useful in understanding the RAAS during pregnancy.

### Supplementary Information

**Supplementary Material 1.**

**Supplementary Material 2.**

## Data Availability

The dataset generated and analyzed during the current study are available from the corresponding author on reasonable request.

## Abbreviations

ANGII: angiotensin II
APRC: active plasma renin concentration
AT_1_-AA: agonistic autoantibodies to the angiotensin II type 1 receptor
AT_1_R: angiotensin II type 1 receptor
CI: confidence interval
FGR: fetal growth restriction
GA: gestational age
IQR: interquartile range
PE: pre-eclampsia
PIH: pregnancy induced hypertension
QUIPS: quality in prognosis studies
RAAS: renin-angiotensin-aldosterone system
REML: restricted maximum likelihood
ROM: ratio of the mean
SD: standard deviation
SE: standard error
SGA: small for gestational age

## References

[1] Boulpaep EL. Regulation of Arterial Pressure and Cardiac Output. Medical Physiology. 3 ed: Elsevier; 2017. p. 533–55.el.

[2] Mulder EG, de Haas S, Mohseni Z, Schartmann N, Abo Hasson F, Alsadah F (2022). Cardiac output and peripheral vascular resistance during normotensive and hypertensive pregnancy - a systematic review and meta-analysis. BJOG..

[3] de Haas S, Ghossein-Doha C, van Kuijk SM, van Drongelen J, Spaanderman ME (2017). Physiological adaptation of maternal plasma volume during pregnancy: a systematic review and meta-analysis. Ultrasound Obstet Gynecol..

[4] Lopes van Balen VA, van Gansewinkel TAG, de Haas S, Spaan JJ, Ghossein-Doha C, van Kuijk SMJ (2019). Maternal kidney function during pregnancy: systematic review and meta-analysis. Ultrasound Obstet Gynecol..

[5] Nicholson EC, Gallery EDM, Brown MA, Ross MR, Jones M (1987). Renin activation in Normal and hypertensive human pregnancy. Clin Experiment Hyperten Part B: Hyperten Pregnancy..

[6] Skinner SL, Cran EJ, Gibson R, Taylor R, Walters WA, Catt KJ (1975). Angiotensins I and II, active and inactive renin, renin substrate, renin activity, and angiotensinase in human liquor amnii and plasma. Am J Obstet Gynecol..

[7] Spaanderman M, Ekhart T, van Eyck J, de Leeuw P, Peeters L (2001). Preeclampsia and maladaptation to pregnancy: a role for atrial natriuretic peptide?. Kidney Int..

[8] Moher D, Shamseer L, Clarke M, Ghersi D, Liberati A, Petticrew M (2015). Preferred reporting items for systematic review and meta-analysis protocols (PRISMA-P) 2015 statement. Syst Rev..

[9] Booth A, Clarke M, Dooley G, Ghersi D, Moher D, Petticrew M, et al. The nuts and bolts of PROSPERO: an international prospective register of systematic reviews. System Rev. 2012;1(1) 10.1186/2046-4053-1-2 PMC3348673 22587842

[10] Rohatgi A. WebPlotDigitizer: version 4.6 2022. Available from: https://automeris.io/WebPlotDigitizer.

[11] Hayden JA, van der Windt DA, Cartwright JL, Côté P, Bombardier C (2013). Assessing bias in studies of prognostic factors. Ann Intern Med..

[12] Higgins JPT, Li T, Deeks JJ. Chapter 6: Choosing effect measures and computing estimates of effect. In: Higgins JPT, Thomas J, Chandler J, Cumpston M, Li T, Page MJ, Welch VA, editors. Cochrane Handbook for Systematic Reviews of Interventions version 6.4 (updated August 2023). Cochrane. 2023. Available from www.training.cochrane.org/handbook.

[13] Hozo SP, Djulbegovic B, Hozo I (2005). Estimating the mean and variance from the median, range, and the size of a sample. BMC Med Res Methodol..

[14] IntHout J, Ioannidis JP, Borm GF (2014). The Hartung-Knapp-Sidik-Jonkman method for random effects meta-analysis is straightforward and considerably outperforms the standard DerSimonian-Laird method. BMC Med Res Methodol..

[15] Higgins JP, Thompson SG, Deeks JJ, Altman DG (2003). Measuring inconsistency in meta-analyses. BMJ..

[16] Egger M, Davey Smith G, Schneider M, Minder C (1997). Bias in meta-analysis detected by a simple, graphical test. BMJ..

[17] R Core Team (2022). R: a language and environment for statistical computing.

[18] Balduzzi SRG (2019). Schwarzer G how to perform a meta-analysis with R: a practical tutorial. Evid Based Ment Health..

[19] Sera F, Armstrong B, Blangiardo M, Gasparrini A (2019). An extended mixed-effects framework for meta-analysis. Stat Med..

[20] Al Kadi H, Nasrat H, Broughton PF (2005). A prospective, longitudinal study of the renin-angiotensin system, prostacyclin and thromboxane in the first trimester of normal human pregnancy: association with birthweight. Hum Reprod..

[21] Baker PN, Broughton Pipkin F, Symonds EM (1992). Longitudinal study of platelet angiotensin II binding in human pregnancy. Clin Sci (Lond)..

[22] Brown MA, Reiter L, Rodger A, Whitworth JA (1994). Impaired renin stimulation in pre-eclampsia. Clin Sci (Lond)..

[23] Brown MA, Thou ST, Whitworth JA (1995). Stimulation of aldosterone by ACTH in normal and hypertensive pregnancy. Am J Hypertens.

[24] Brown MA, Zammit VC, Adsett D (1990). Stimulation of active renin release in normal and hypertensive pregnancy. Clin Sci (Lond)..

[25] Brown MA, Zammit VC, Mitar DA, Whitworth JA (1992). Renin-aldosterone relationships in pregnancy-induced hypertension. Am J Hypertens..

[26] Brown MA, Zammit VC, Mitar DA, Whitworth JA (1993). Control of aldosterone in Normal and hypertensive pregnancy: effects of metoclopramide. Hyperten Pregnancy..

[27] Brown MA, Zammit VC, Whitworth JA (1992). Renal prostacyclin, renin and glomerular filtration in pregnancy-induced hypertension. Clin Experiment Hyperten Part B: Hyperten Pregnancy..

[28] Derkx FH, Alberda AT, de Jong FH, Zeilmaker FH, Makovitz JW, Schalekamp MA (1987). Source of plasma prorenin in early and late pregnancy: observations in a patient with primary ovarian failure. J Clin Endocrinol Metab..

[29] Jarvis SS, Shibata S, Bivens TB, Okada Y, Casey BM, Levine BD (2012). Sympathetic activation during early pregnancy in humans. J Physiol..

[30] Langer B, Grima M, Coquard C, Bader AM, Schlaeder G, Imbs JL (1998). Plasma active renin, angiotensin I, and angiotensin II during pregnancy and in preeclampsia. Obstet Gynecol..

[31] Pedersen EB, Christensen NJ, Christensen P, Johannesen P, Kornerup HJ, Kristensen S (1982). Prostaglandins, catecholamines, renin and aldosterone during hypertensive and normotensive pregnancy. Clin Exp Hypertens A..

[32] Skinner SL, Lumbers ER, Symonds EM (1972). Analysis of changes in the renin-angiotensin system during pregnancy. Clin Sci..

[33] Spaan JJ, Bowyer L, Lazzaro VA, McCrohon J, Brown MA (2013). Maternal hemodynamics influence fetal hemodynamics in normal and hypertensive pregnancy. Pregnancy Hypertens..

[34] Thomsen JK, Fogh-Andersen N, Jaszczak P, Giese J (1993). Atrial natriuretic peptide (ANP) decrease during normal pregnancy as related to hemodynamic changes and volume regulation. Acta Obstet Gynecol Scand..

[35] Lewandowski KC, Tadros-Zins M, Horzelski W, Krekora M, Lewinski A (2023). Renin, aldosterone, and cortisol in pregnancy-induced hypertension. Exp Clin Endocrinol Diabetes..

[36] Pedersen EB, Rasmussen AB, Johannesen P, Kornerup HJ, Kristensen S, Lauritsen JG (1982). The renin-aldosterone system in pre-eclampsia, essential and transient hypertension during pregnancy, and normotensive pregnant and non-pregnant control subjects. Acta Endocrinol..

[37] Lumbers ER, Pringle KG (2014). Roles of the circulating renin-angiotensin-aldosterone system in human pregnancy. Am J Physiol Regul Integr Comp Physiol..

[38] Peti-Peterdi J, Harris RC (2010). Macula densa sensing and signaling mechanisms of renin release. J Am Soc Nephrol..

[39] Irani RA, Xia Y (2011). Renin angiotensin signaling in normal pregnancy and preeclampsia. Semin Nephrol..

[40] Lumbers ER, Delforce SJ, Arthurs AL, Pringle KG (2019). Causes and consequences of the dysregulated maternal renin-angiotensin system in preeclampsia. Front Endocrinol (Lausanne)..

[41] Symonds EM (1981). The renin-angiotensin system in pregnancy. Obstet Gynecol Annu..

[42] Campbell DJ, Nussberger J, Stowasser M, Danser AH, Morganti A, Frandsen E (2009). Activity assays and immunoassays for plasma renin and prorenin: information provided and precautions necessary for accurate measurement. Clin Chem..

