# Supplementary material for: Active plasma renin concentration throughout healthy and complicated pregnancy: a systematic review and meta-analysis
Source: Reprod Biol Endocrinol. 2024 Mar 7;22:29. doi: 10.1186/s12958-024-01200-2 (PMC10918957; doi:10.1186/s12958-024-01200-2)
Supplement: Supplementary file 1 — Supplementary Material 1. [file 12958_2024_1200_MOESM1_ESM.docx]

Appendices

[Appendix S1: Search strategy 3](#_Toc152945631)

[Appendix S2: Study characteristics 6](#_Toc152945632)

[S2.1 Study characteristics of included studies with healthy pregnancies 6](#_Toc152945633)

[S2.2 Study characteristics of included studies with complicated pregnancies 8](#_Toc152945634)

[S2.3 Method and circumstances of renin measurement of the included studies. 10](#_Toc152945635)

[Appendix S3: funnel plot 12](#_Toc152945636)

[Appendix S4: Sensitivity analyses of APRC in healthy pregnancy 13](#_Toc152945637)

[Figure S4.1 13](#_Toc152945638)

[Figure S4.2. 13](#_Toc152945639)

[Figure S4.3 13](#_Toc152945640)

[Figure S4.4 13](#_Toc152945641)

[Figure S4.5 14](#_Toc152945642)

[Figure S4.6 14](#_Toc152945643)

[Figure S4.7 14](#_Toc152945644)

[Figure S4.8 14](#_Toc152945645)

[Figure S4.9 15](#_Toc152945646)

[Figure S4.10 15](#_Toc152945647)

[Figure S4.11 15](#_Toc152945648)

[Figure S4.12 15](#_Toc152945649)

[Figure S4.13 16](#_Toc152945650)

[Appendix S5: Sensitivity analyses of APRC in complicated pregnancy 16](#_Toc152945651)

[Figure S5.1 16](#_Toc152945652)

[Figure S5.2 16](#_Toc152945653)

[Figure S5.3 16](#_Toc152945654)

[Figure S5.4 17](#_Toc152945655)

[Figure S5.5 17](#_Toc152945656)

# Appendix S1: Search strategy

| # | Component | Pubmed | Embase |
| --- | --- | --- | --- |
| 1 | Pregnancy | "pregnancy"[Mesh] OR pregnancy[tiab] OR pregnancies[tiab] OR pregnant[tiab] OR gestation[tiab] OR gestations[tiab] OR gestational[tiab] OR "gravidity"[Mesh] OR gravidity[tiab] OR gravidities[tiab] OR gravid[tiab] | exp pregnancy/ OR pregnancy.ti,ab,kw. OR pregnancies.ti,ab,kw. OR pregnant.ti,ab,kw. OR gestation.ti,ab,kw. OR gestations.ti,ab,kw. OR gestational.ti,ab,kw. OR gravidity.ti,ab,kw. OR gravidities.ti,ab,kw. OR gravid.ti,ab,kw. |
| 2 | PIH | "hypertension, pregnancy-induced"[Mesh] OR "pregnancy induced hypertension"[tiab] OR "pregnancy associated hypertension"[tiab] OR PIH[tiab] OR "hypertensive pregnancy"[tiab] OR "pregnancy hypertension"[tiab] OR "gestational hypertension"[tiab] | exp maternal hypertension/ OR pregnancy induced hypertension.ti,ab,kw. OR pregnancy associated hypertension.ti,ab,kw. OR PIH.ti,ab,kw. OR hypertensive pregnancy.ti,ab,kw. OR pregnancy hypertension.ti,ab,kw. OR gestational hypertension.ti,ab,kw. |
| 3 | PE | "pre-eclampsia"[Mesh] OR "pre-eclampsia"[tiab] OR preeclampsia[tiab] OR preeclamptic[tiab] OR pre-eclamptic[tiab] OR PE[tiab] OR "eclampsia"[Mesh] OR eclampsia[tiab] OR eclampsias[tiab] OR eclamptic[tiab] OR toxemia[tiab] OR toxemias[tiab] | exp eclampsia and preeclampsia/  OR "pre-eclampsia".ti,ab,kw. OR preeclampsia.ti,ab,kw. OR preeclamptic.ti,ab,kw. OR pre-eclamptic.ti,ab,kw. OR PE.ti,ab,kw. OR eclampsia.ti,ab,kw. OR eclampsias.ti,ab,kw. OR eclamptic.ti,ab,kw. OR toxemia.ti,ab,kw. OR toxemias.ti,ab,kw. |
| 4 | HELLP syndrome | "HELLP syndrome"[Mesh] OR "HELLP"[tiab] OR "Hemolysis, Elevated Liver Enzymes, Lowered Platelets"[tiab]) | exp HELLP syndrome/ OR HELLP.ti,ab,kw. OR Hemolysis, Elevated Liver Enzymes, Lowered Platelets".ti,ab,kw. OR hemolysis, elevated liver enzymes, low platelet.ti,ab,kw. OR hemolysis, elevated liver enzymes, low platelets.ti,ab,kw. |
| 5 | Gestational diabetes | "Diabetes, Gestational"[Mesh] OR "pregnancy induced diabetes"[tiab] OR "gestational diabetes"[tiab] OR "diabetes gravidarum"[tiab] | exp pregnancy diabetes mellitus/ OR pregnancy induced diabetes.ti,ab,kw. OR gestational diabetes.ti,ab,kw.  OR diabetes gravidarum.ti,ab,kw. |
| 6 | FGR | "fetal Growth Retardation"[Mesh] OR "fetal growth retardation"[tiab] OR "fetal growth restriction"[tiab] OR FGR[tiab] OR "intrauterine growth retardation"[tiab] OR "intrauterine growth restriction"[tiab] OR IUGR[tiab] | exp intrauterine growth retardation/ OR fetal growth retardation.ti,ab,kw. OR "fetal growth restriction.ti,ab,kw. OR FGR.ti,ab,kw. OR intrauterine growth retardation.ti,ab,kw. OR intrauterine growth restriction.ti,ab,kw. OR IUGR.ti,ab,kw. |
| 7 | SGA | "Infant, Small for Gestational Age"[Mesh] OR "small for gestational age"[tiab] OR SGA[tiab] | exp small for date infant/ OR small for gestational age.ti,ab,kw. OR SGA.ti,ab,kw. |
| 8 | RAAS | "renin-Angiotensin System"[Mesh] OR Renin-Angiotensin System[tiab] OR Renin Angiotensin Aldosterone System[tiab] | exp renin angiotensin aldosterone system / OR Renin-Angiotensin System.ti,ab,kw. OR Renin Angiotensin Aldosterone System.ti,ab,kw. |
| 9 | Renin | "renin"[Mesh] OR renin [tiab] OR Angiotensin Forming Enzyme[tiab] OR Angiotensinogenase[tiab]) | exp renin/ OR kidney renin/ OR renin.ti,ab,kw. OR Angiotensin Forming Enzyme.ti,ab,kw. OR Angiotensinogenase.ti,ab,kw. |

Combined as: (#1 OR #2 OR #3 OR #4 OR #5 OR #6 OR #7) AND (#8 OR #9)

# Appendix S2: Study characteristics

## S2.1 Study characteristics of included studies with healthy pregnancies

**Table S2.1.** Study characteristics of included studies on plasma active renin measurements in reference (Ref) and healthy pregnancy (Preg) group.

| **Study** | **Subjects (N)** | | **Age**  **(years)** | | **Weight**  **(kg)** | | **Height**  **(cm)** | | **Blood pressure**  **(mmHg)** | | **Parity/gravidity (N)** | | | | | | **Type of reference group** | **GA at renin measurement (weeks)** | **Phase of MC at renin measurement of reference**  **Group (N)** | |  |
| --- | --- | --- | --- | --- | --- | --- | --- | --- | --- | --- | --- | --- | --- | --- | --- | --- | --- | --- | --- | --- | --- |
|  |  |  |  |  |  |  |  |  |  | | **Nulli-** | | **Primi-** | | **Multi-** | |  |  | **F** | **L** |  |
|  | **Ref** | **Preg** | **Ref** | **Preg** | **Ref** | **Preg** | **Ref** | **Preg** | **Ref** | **Preg** | **Ref** | **Preg** | **Ref** | **Preg** | **Ref** | **Preg** |  |  |  |  |  |
| Al Kadi  (2005)^20^ | **10** | **12** | **25** | **25** | **-** | **-** | **-** | **-** | **-** | **-** | **10** | **12** | **-** | **-** | **-** | **-** | **PC** | **6** | **10** | **-** |  |
| **Baker (1992)^21^** | **30** | **30** | **-** | **-** | **-** | **-** | **-** | **-** | **-** | **-** | **30** | **30** | **-** | **-** | **-** | **-** | **PP (6 and 12 weeks)** | **11, 18, 28 and 36** | **-** | **-** |  |
| **Brown (1990)^24^** | **19** | **13** | **27** | **24** | **57.3** | **71.3** | **165** | **162** | **105/68** | **108/63** | **-** | **-** | **-** | ***13*** | **-** | **-** | **NP** | **35** | **-** | **-** |  |
|  | **19** | **15** | **27** | **24** | **57.3** | **69.0** | **165** | **162** | **105/68** | **104/66** | **-** | **-** | **-** | ***15*** | **-** | **-** | **NP** | **35** | **-** | **-** |  |
| **Brown**  **(1992a)^25^** | **80** | **83** | **26** | **25** | **60** | **71.9** | **-** | **-** | **109/68** | **109/67** | **-** | **-** | **-** | **-** | **-** | **-** | **NP** | **35** | **-** | **-** |  |
| **Brown**  **(1992b)^27^** | **20** | **37** | **25** | **25** | **60** | **74.1** | **167** | **164** | **-** | **-** | **-** | **37** | **-** | **-** | **-** | **-** | **NP** | **35** | **-** | **-** |  |
| **Brown**  **(1993)^26^** | **6** | **6** | **25** | **27** | **F=58.1**  **L=55.5** | **82.2** | **164** | **161** | **F=109/66**  **L=105/65** | **107/63** | **-** | **-** | **-** | ***6*** | **-** | **-** | **NP** | **36** | **6** | **6** |  |
| **Brown (1994)^22^** | **16** | **16** | **-** | **-** | **-** | **-** | **-** | **-** | **103/65** | **107/76** | **-** | **-** | **-** | **-** | **-** | **-** | **NP** | **Third trimester** | **-** | **-** |  |
| **Brown (1995)^23^** | **10** | **9** | **26** | **27** | **61** | **82** | **159** | **163** | **101/68** | **107/57** | **-** | **-** | **-** | **-** | **-** | **-** | **NP** | **32** | **-** | **-** |  |
| **Derkx**  **(1987)^28^** | **9** | **9** | **28-38** | **28-38** | **-** | **-** | **-** | **-** | **-** | **-** | **-** | **-** | **-** | **-** | **-** | **-** | **PP**  **(6-8weeks)** | **8 and 36** | **-** | **-** |  |
| Jarvis  (2012)^29^ | **11** | **11** | **29** | **30** | **63.2** | **63.3** | **162.1** | **161.8** | **-** | **-** | **-** | **-** | **-** | **-** | **-** | **-** | **PC** | **6.2** | **-** | **11** |  |
| Langer (1998)^30^ | **7** | **7** | **28.3** | **28.3** | **-** | **-** | **-** | **-** | **D=76** | **D=76** | **3** | **3** | **-** | **-** | **-** | **-** | **PP**  **(6 weeks)** | **33.5** | **-** | **-** |  |
| Lewandow-ski (2023)^35^ | **22** | **70** | **33**  **(19-44)** | **31**  **(22-42)** | **-** | **-** | **-** | **-** | **110-120**  **/60-80** | **110-120 /60-80** | **-** | **-** | **-** | **-** | **-** | **-** | **NP** | **32.38+-4.25** | **-** | **-** |  |
| Nicholson  (1987)^5^ | **14** | **20** | **19-40** | **19-40** | **-** | **-** | **-** | **-** | **-** | **-** | **-** | **-** | **-** | ***20*** | **-** | **-** | **NP** | **30-36** | **-** | **14** |  |
| **Pedersen (1982)^31^** | **18** | **18** | **26** | **26** | **-** | **-** | **-** | **-** | **108/64** | **108/64** | **-** | **-** | **-** | **15** | **-** | **3** | **PP**  **(3 months)** | **40** | **-** | **-** |  |
| **Skinner (1972)^32^** | **9** | **34** | **-** | **-** | **-** | **-** | **-** | **-** | **-** | **-** | **-** | **-** | **-** | **-** | **-** | **-** | **NP** | **4-40** | **-** | **9** | |
| Spaan (2013)^33^ | **18** | **25^c^** | **29** | **29** | **73.1** | **73.1** | **-** | **-** | **MAP=79** | **MAP=81** | **-** | **-** | **-** | **-** | **10** | **10** | **PP**  **(4-10 months)** | **33^5/7^** | **-** | **-** | |
| Spaander-man (2001)^7^ | **10** | **10** | **31** | **31** | **-** | **-** | **168** | **168** | **MAP=79** | **MAP=79** | **-** | **-** | **70%** | **70%** | **-** | **-** | **PC** | **5 and 7** | **10** | **0** | |
| **Thomsen**  **(1993)^34^** | **40** | **40** | **25** | **25** | **-** | **-** | **-** | **-** | **-** | **-** | **-** | **-** | ***40*** | ***40*** | **-** | **-** | **PP**  **(12 weeks)** | **28, 32, 36 and 38** | **-** | **-** | |

Parity and gravidity are reported in normal font line and italic, respectively. GA=gestational age, MC=menstrual cycle; F=follicular phase; L=luteal phase; D=diastolic blood pressure; MAP=mean arterial pressure; NP=nonpregnant; PC=preconception; PP=postpartum.

## S2.2 Study characteristics of included studies with complicated pregnancies

**Table S2.2.** Study characteristics of included studies on plasma renin measurements in reference (Ref) and complicated pregnancy (Preg) group.

| **Study** | **Subjects (n)** | | **Age**  **(years)** | | **Weight**  **(kg)** | | **Height (cm)** | | **Blood**  **pressure**  **(mmHg)** | | **Parity/gravidity** | | | | | | **Ref group** | **GA at renin measurement (weeks)** | **Phase of MC at renin measurement of reference**  **group (n)** | |
| --- | --- | --- | --- | --- | --- | --- | --- | --- | --- | --- | --- | --- | --- | --- | --- | --- | --- | --- | --- | --- |
|  |  |  |  |  |  |  |  |  |  | | **Nulli-** | | **Primi-** | | **Multi-** | |  |  | **F** | **L** |
|  | **Ref** | **Preg** | **Ref** | **Preg** | **Ref** | **Preg** | **Ref** | **Preg** | **Ref** | **Preg** | **Ref** | **Preg** | **Ref** | **Preg** | **Ref** | **Preg** |  |  |  |  |
| **Brown**  **(1990)^24^** | **19** | **10** | **27** | **31** | **57.3** | **72.2** | **165** | **158** | **105/68** | **134/91** | **-** | **-** | **-** | **-** | **-** | **-** | **NP** | **35** | **-** | **-** |
| **Brown**  **(1992a)^25^** | **80** | **50** | **26** | **28** | **60** | **78.6** | **-** | **-** | **109/68** | **131/90** | **-** | **50** | **-** | **-** | **-** | **-** | **NP** | **36** | **-** | **-** |
| **Brown**  **(1992b)^27^** | **20** | **21*** | **25** | **26** | **60** | **81.8** | **167** | **164** | **-** | **-** | **-** | **21** | **-** | **-** | **-** | **-** | **NP** | **37** | **-** | **-** |
|  | **20** | **20**^†^ | **25** | **31** | **60** | **75.4** | **167** | **164** | **-** | **-** | **-** | **20** | **-** | **-** | **-** | **-** | **NP** | **35** | **-** | **-** |
| **Brown**  **(1993)^26^** | **6** | **9** | **25** | **29** | **F=58.1**  **F=55.5** | **79.2** | **164** | **163** | **F=109/66**  **L=105/65** | **136/99** | **-** | **-** | **-** | ***9*** | **-** | **-** | **NP** | **34** | **6** | **6** |
| **Brown (1994)^22^** | **16** | **9** | **-** | **-** | **-** | **-** | **-** | **-** | **103/65** | **140/96** | **-** | **-** | **-** | **-** | **-** | **-** | **NP** | **Third trimester** | **-** | **-** |
| **Brown (1995)^23^** | **10** | **9** | **26** | **27** | **61** | **87** | **159** | **163** | **101/68** | **140/93** | **-** | **-** | **-** | **-** | **-** | **-** | **NP** | **35** | **-** | **-** |
| Langer (1998)^30^ | **7** | **8**^‡^ | **28.3** | **28.9** | **-** | **-** | **-** | **-** | **D=76** | **D=106** | **3** | **6** | **-** | **-** | **-** | **-** | **Healthy PP**  **(6 weeks)** | **35.4** | **-** | **-** |
| **Lewandow-ski (2023)^35^** | **22** | **62** | **33**  **(19-44)** | **30**  **(19-44)** | **-** | **-** | **-** | **-** | **110-120**  **/60-80** | **148-160**  **/90-100** | **-** | **-** | **-** | **-** | **-** | **-** | **NP** | **32.2+-3.96** | **-** | **-** |
| **Pedersen**  **(1982)^31^** | **19** | **15** | **23** | **28** | **-** | **-** | **-** | **-** | **109/66** | **153/103** | **-** | **-** | **-** | **11** | **-** | **4** | **NP** | **36** | **10** | **9** |
| Spaan (2013)^33^ | **16** | **21**^§^ | **30** | **30** | **84** | **84** | **-** | **-** | **MAP=90** | **MAP=105** | **-** | **-** | **-** | **-** | **10** | **10** | **PP**  **(4-10 months)** | **37^2/7^** | **-** | **-** |
|  | **7** | **10**^¶^ | **30** | **30** | **81.8** | **81.8** | **-** | **-** | **MAP=87** | **MAP=106** | **-** | **-** | **-** | **-** | **5** | **5** | **PP**  **(4-10 months)** | **36^0/7^** | **-** | **-** |

Parity and gravidity are reported in normal font line and italic, respectively. GA=gestational age, MC=menstrual cycle; F=follicular phase; L=luteal phase; D=diastolic blood pressure; MAP=mean arterial pressure; NP=nonpregnant; PC=preconception; PP=postpartum. * Mild PIH, ^†^ Severe PIH, ^‡^ Six women with SGA baby, ^§^ Gestational hypertension, ^¶^ Preeclampsia

## S2.3 Method and circumstances of renin measurement of the included studies.

**Table S2.3.** Method and circumstances of renin measurement of the included studies.

| References | Method of measurement | Posture during blood sampling | Time of blood sampling (h) | Fasting | Diet |
| --- | --- | --- | --- | --- | --- |
| Al Kadi (2005)^20^ | Activity assay | Seated with arm supported | - | - |  |
| Baker (1992)^21^ | Activity assay | Semi-prone position | Morning | No | - |
| Brown (1990)^24^ | Activity assay | Left lateral recumency  (20 min) | 09:00-12:00 | - | - |
| **Brown (1992a)^25^** | Activity assay | Left lateral recumbency  (30-60min) | 09:00-11:00 | - | Free diet |
| **Brown (1992b)^27^** | Activity assay | Left lateral recumbency  (after 25-30 resting) | 08:00-12:00 | - | - |
| **Brown (1993)^26^** | Activity assay | Left lateral recumbency (for 60 min) after 10 min of rest. | 09:30-12:00 | - | Ad-libitum diet |
| Brown (1994)^22^ | Activity assay | Left lateral recumbency (1h) | Morning | - | Ad libitum diet  Oral water load (500ml) in 30 min |
| **Brown (1995)^23^** | Activity assay | Lateral recumbency  (10 min) | - | - | -NP and HP: 100 mmol sodium/day for 4 days prior to study  -PE: free diet |
| Derkx (1987)^28^ | Activity assay and immunoassay | Left lateral recumbency (30 min) | - | - | - |
| Jarvis (2012)^29^ | Direct renin measurement | Supine and 60 degrees upright tilt | - | ≥ 2h after light meal and ≥ 48h last caffeinated or alcoholic beverage | Isocaloric constant diet of 200 mEq sodium |
| Langer (1998)^30^ | Immunoassay (Eria Sanofi Diagnostics Pasteur) | Decubitus (30 min) | 08:00-10:00 | Yes | Unrestrained sodium diet |
| Lewandowksi (2023)^35^ | Immunoassay (Liaison DiaSorin Inc.) | Supine and upright | Supine:  6.00-7.00  Upright:  8.00-9.00 | Yes | Standard hospital diet |
| Nicholson (1987)^5^ | Activity assay | - | - | - |  |
| **Pedersen (1982)^31^** | **Activity assay** | Supine | 09:00 | Yes (8 hours) |  |
| **Skinner (1972)^32^** | Activity assay | Seated | 11:00 | - | Usual diet |
| Spaan (2013)^33^ | - | - | Around the middle of the day | - |  |
| Spaanderman  (2001)^7^ | Immunoradiometric assay technique (Nichols Institute Diagnostics) | Supine | 8:00 | Yes (at least 10 hours before experiment) | One week of standardized sodium intake of 100mmol per day |
| **Thomsen**  **(1993)^34^** | Activity assay | Laid down, tilted a little to the left | 08:00-10:00 | Yes (overnight) | - |

HP=healthy pregnancy; NP=nonpregnant; PE=preeclampsia.

# Appendix S3: Funnel plot


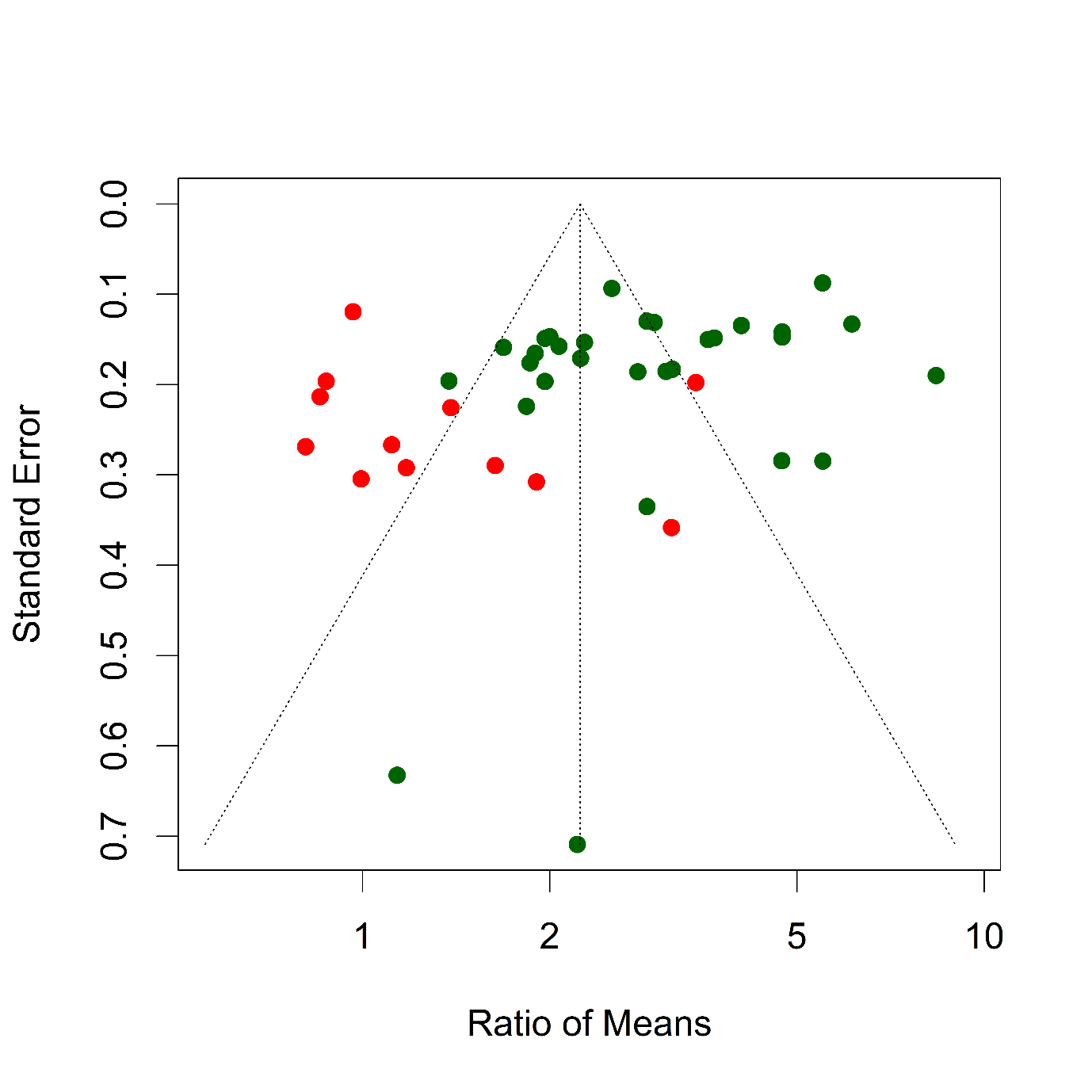


Figure S3. Funnel plot of the ratio of means for active plasma renin concentrations (APRC) during healthy pregnancy. The green data points represent healthy pregnancy; the red data points represent complicated pregnancy.

# Appendix S4: Sensitivity analyses of APRC in healthy pregnancy

## Figure S4.1


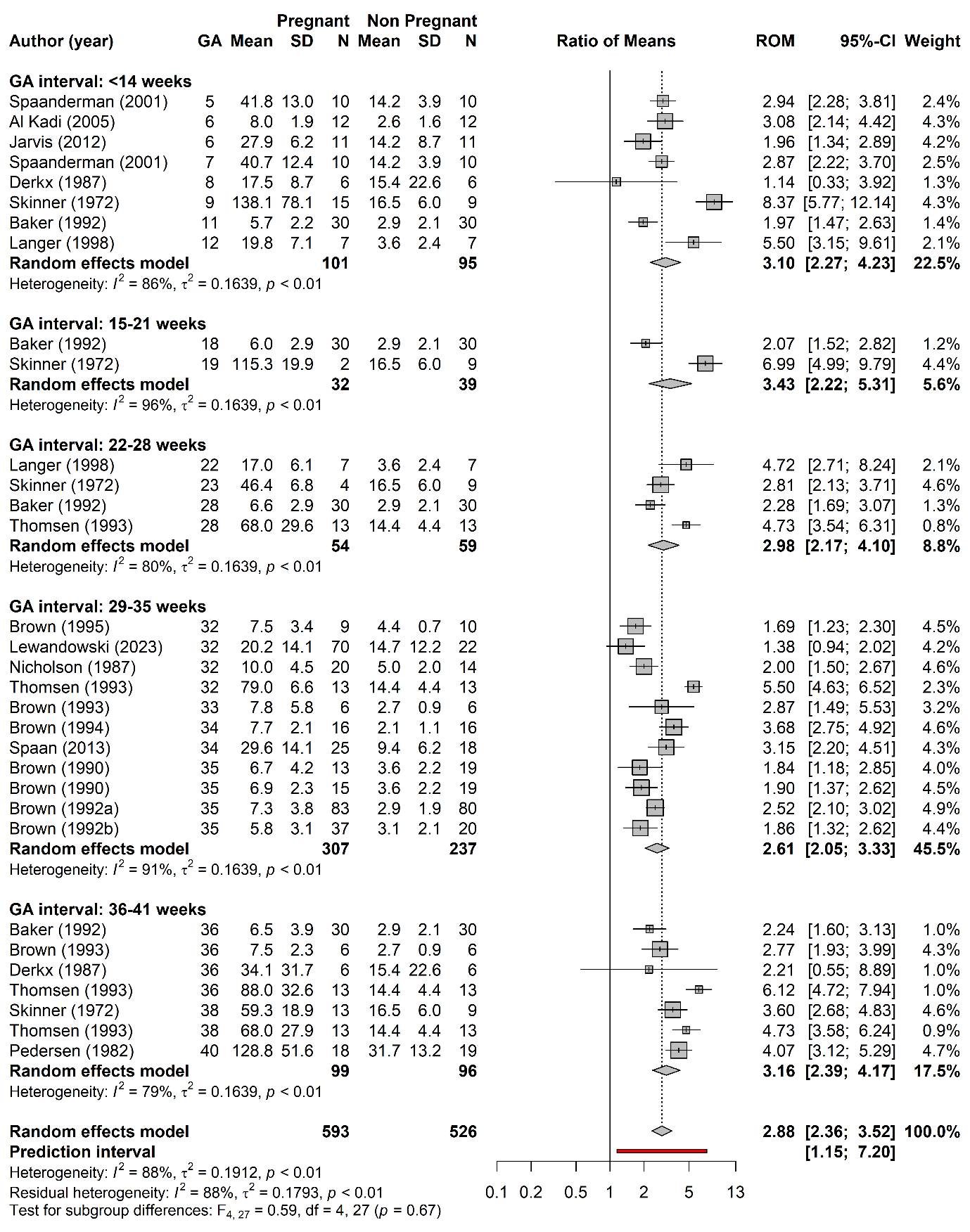


Figure S4.1. Forest plot of the ratio of means (ROM) of the active plasma renin concentrations (APRC) during healthy pregnancy at <14 weeks, 15-21 weeks, 22-28 weeks, 29-35 weeks and 36-41 weeks of gestation compared to reference values in non-pregnancy, preconception or postpartum with no restriction to number of participants. Studies that are reported more than once provided data for different gestational weeks within the same study. Only the first author of each study is given. *GA = gestational age in weeks, SD = standard deviation, CI = confidence interval.*

## Figure S4.2.


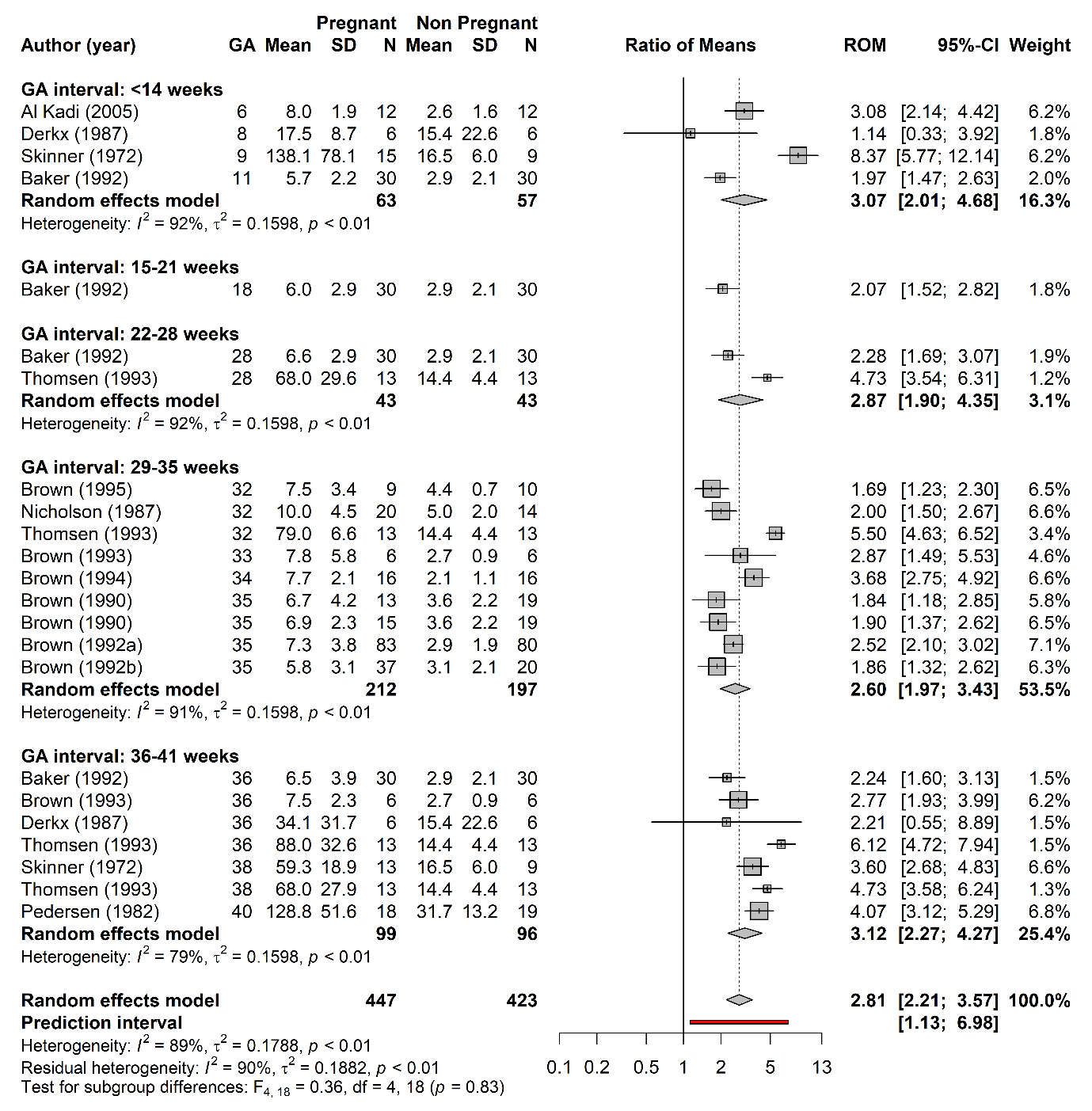


Figure S4.2. Forest plot of the ratio of means (ROM) of the active plasma renin concentrations (APRC) restricted to studies that used an activity assay during healthy pregnancy at <14 weeks, 15-21 weeks, 22-28 weeks, 29-35 weeks and 36-41 weeks of gestation compared to reference values in non-pregnancy, preconception or postpartum. Studies that are reported more than once provide data for different gestational weeks within the same study. Only the first author of each study is given. *GA = gestational age in weeks, SD = standard deviation, CI = confidence interval.*

## Figure S4.3


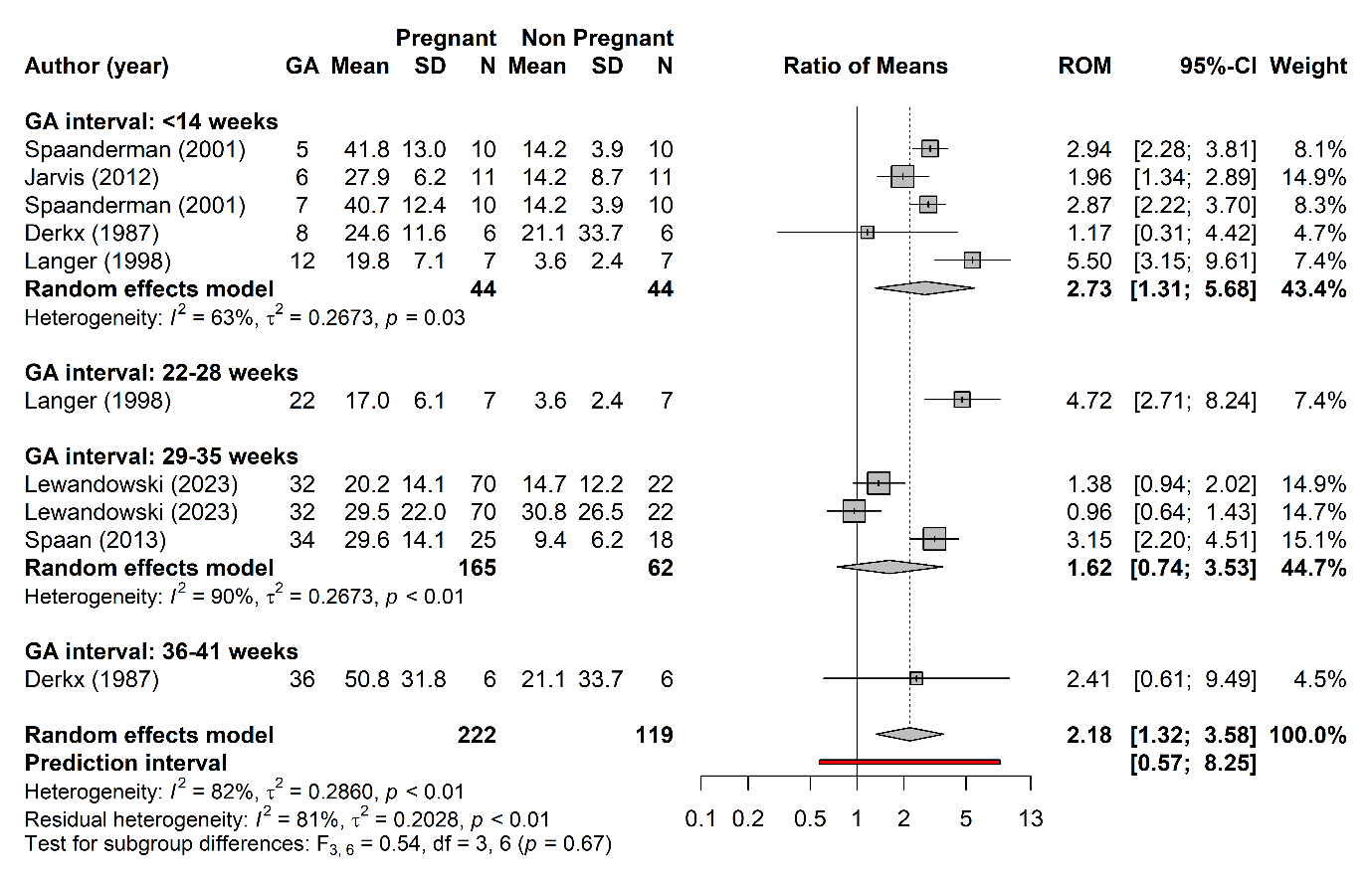


Figure S4.3. Forest plot of the ratio of means (ROM) of the active plasma renin concentrations (APRC) restricted to studies that used an immunoassay during healthy pregnancy at <14 weeks, 15-21 weeks, 22-28 weeks, 29-35 weeks and 36-41 weeks of gestation compared to reference values in non-pregnancy, preconception or postpartum. Studies that are reported more than once provide data for different gestational weeks within the same study. Only the first author of each study is given. *GA = gestational age in weeks, SD = standard deviation, CI = confidence interval.*

## Figure S4.4


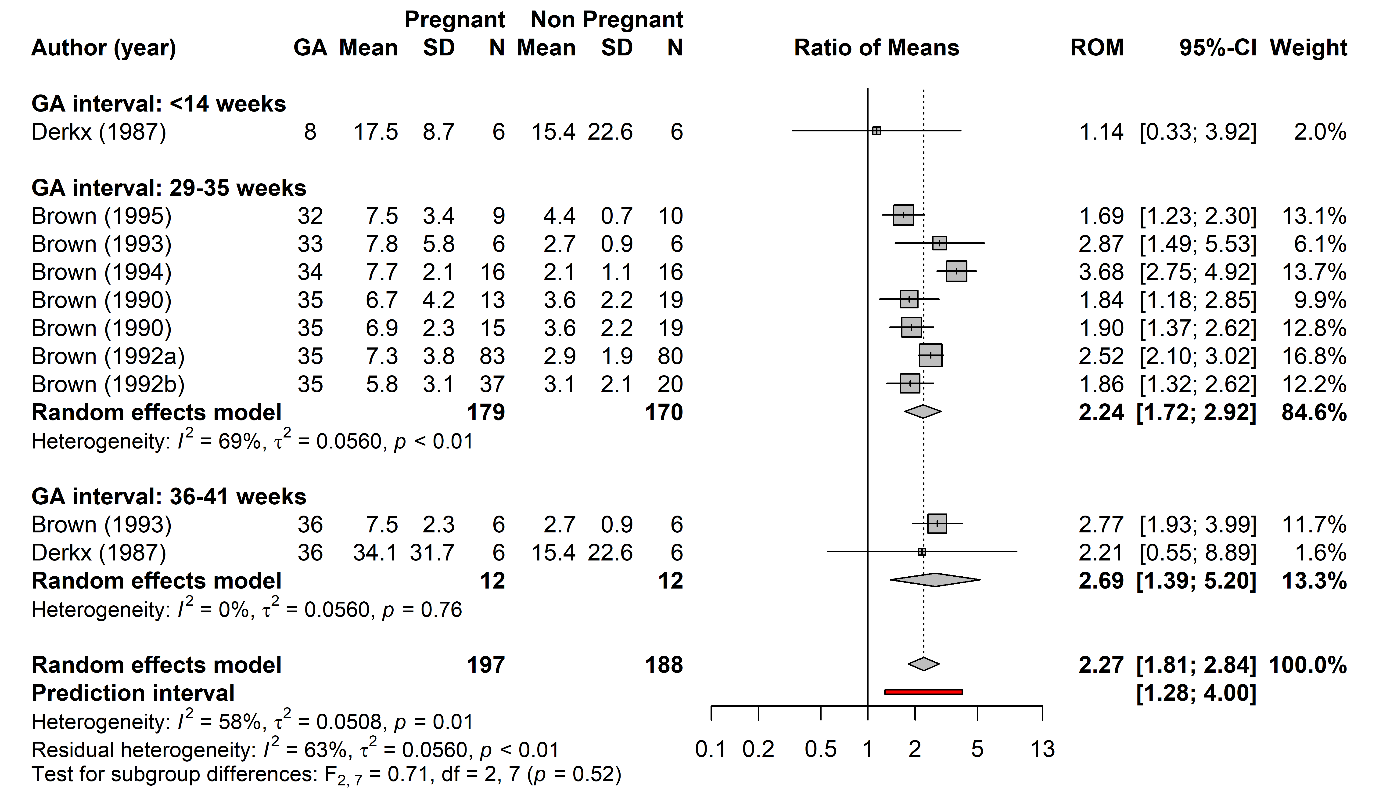


Figure S4.4. Forest plot of the ratio of means (ROM) of the active plasma renin concentrations (APRC) during healthy pregnancy at <14 weeks, 29-35 weeks and 36-41 weeks of gestation compared to reference values in non-pregnancy, preconception or postpartum. The analysis is restricted to studies that collected blood samples to determine renin concentrations in left lateral recumbency position. Studies that are reported more than once provide data for different gestational weeks within the same study. Only the first author of each study is given. *GA = gestational age in weeks, SD = standard deviation, CI = confidence interval.*

## Figure S4.5


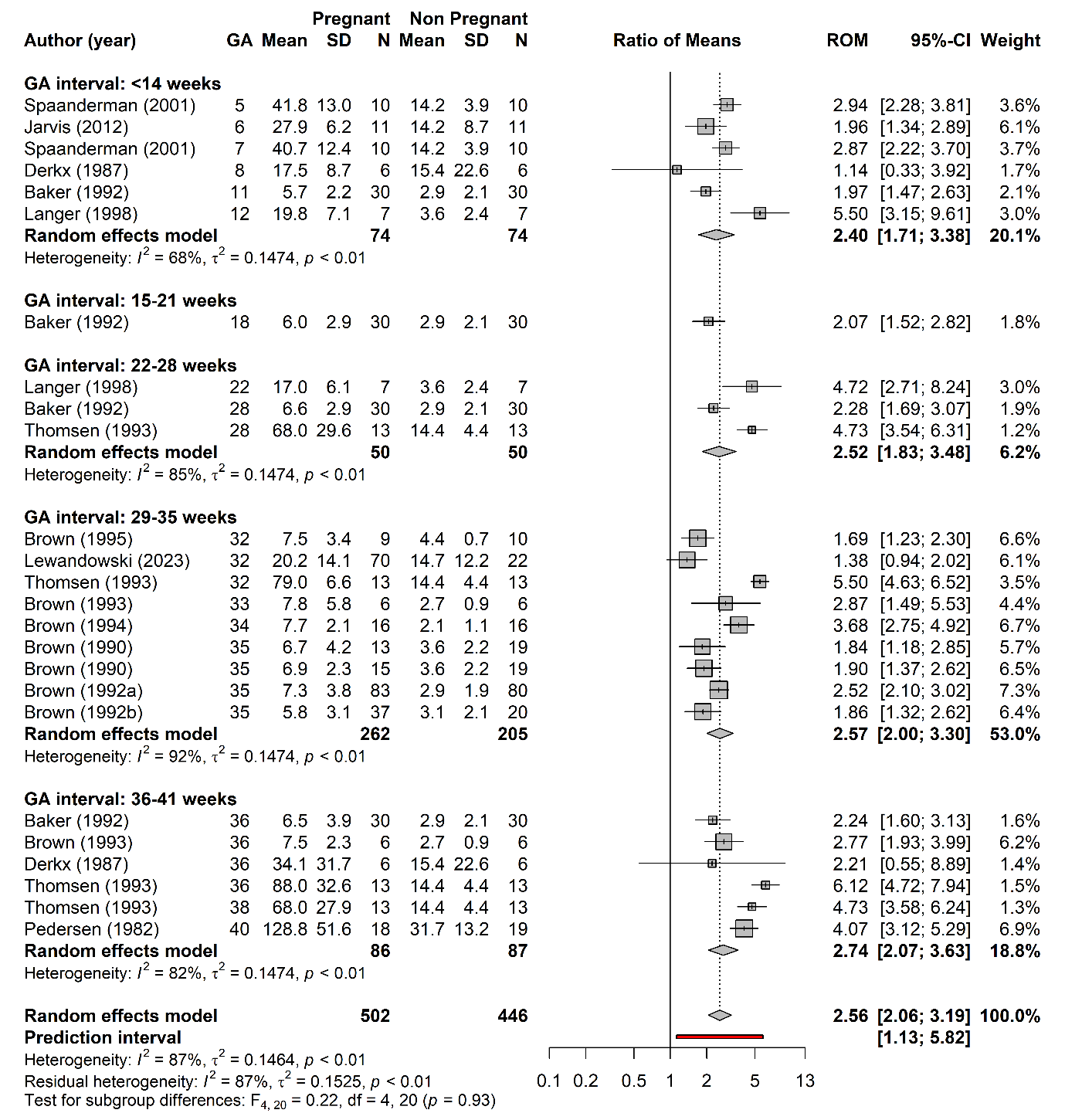


Figure S4.5. Forest plot of the ratio of means (ROM) of the active plasma renin concentrations (APRC) during healthy pregnancy at <14 weeks, 15-21 weeks, 22-28 weeks, 29-35 weeks and 36-41 weeks of gestation compared to reference values in non-pregnancy, preconception or postpartum. The analysis is restricted to studies that collected blood samples to determine renin concentrations in laying position. Studies that are reported more than once provide data for different gestational weeks within the same study. Only the first author of each study is given. *GA = gestational age in weeks, SD = standard deviation, CI = confidence interval.*

## Figure S4.6


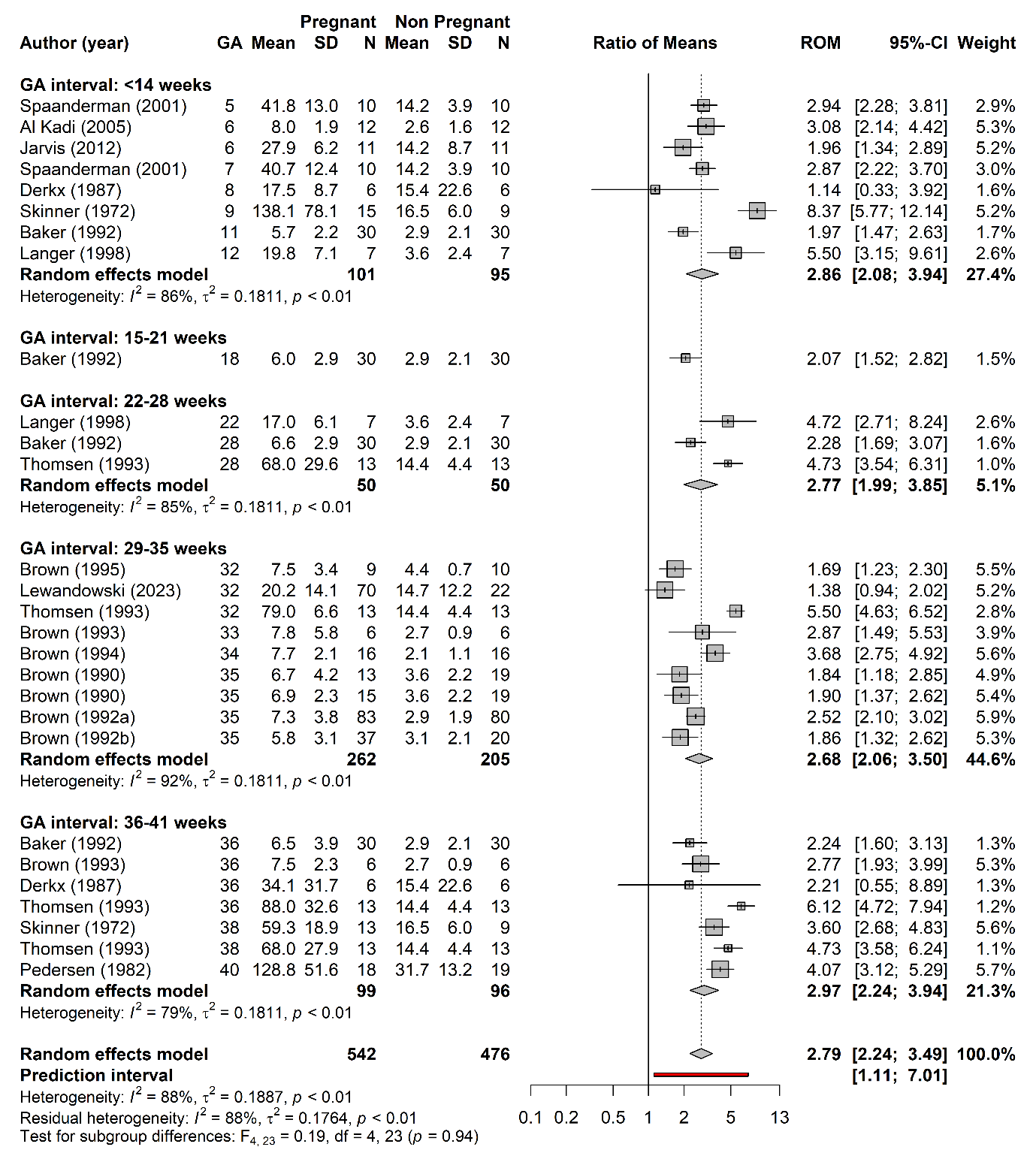


Figure S4.6. Forest plot of the ratio of means (ROM) of the active plasma renin concentrations (APRC) during healthy pregnancy at <14 weeks, 15-21 weeks, 22-28 weeks, 29-35 weeks and 36-41 weeks of gestation compared to reference values in non-pregnancy, preconception or postpartum. The analysis is restricted to studies that collected blood samples to determine renin concentrations in laying or seated position. Studies that are reported more than once provide data for different gestational weeks within the same study. Only the first author of each study is given. *GA = gestational age in weeks, SD = standard deviation, CI = confidence interval.*

## Figure S4.7


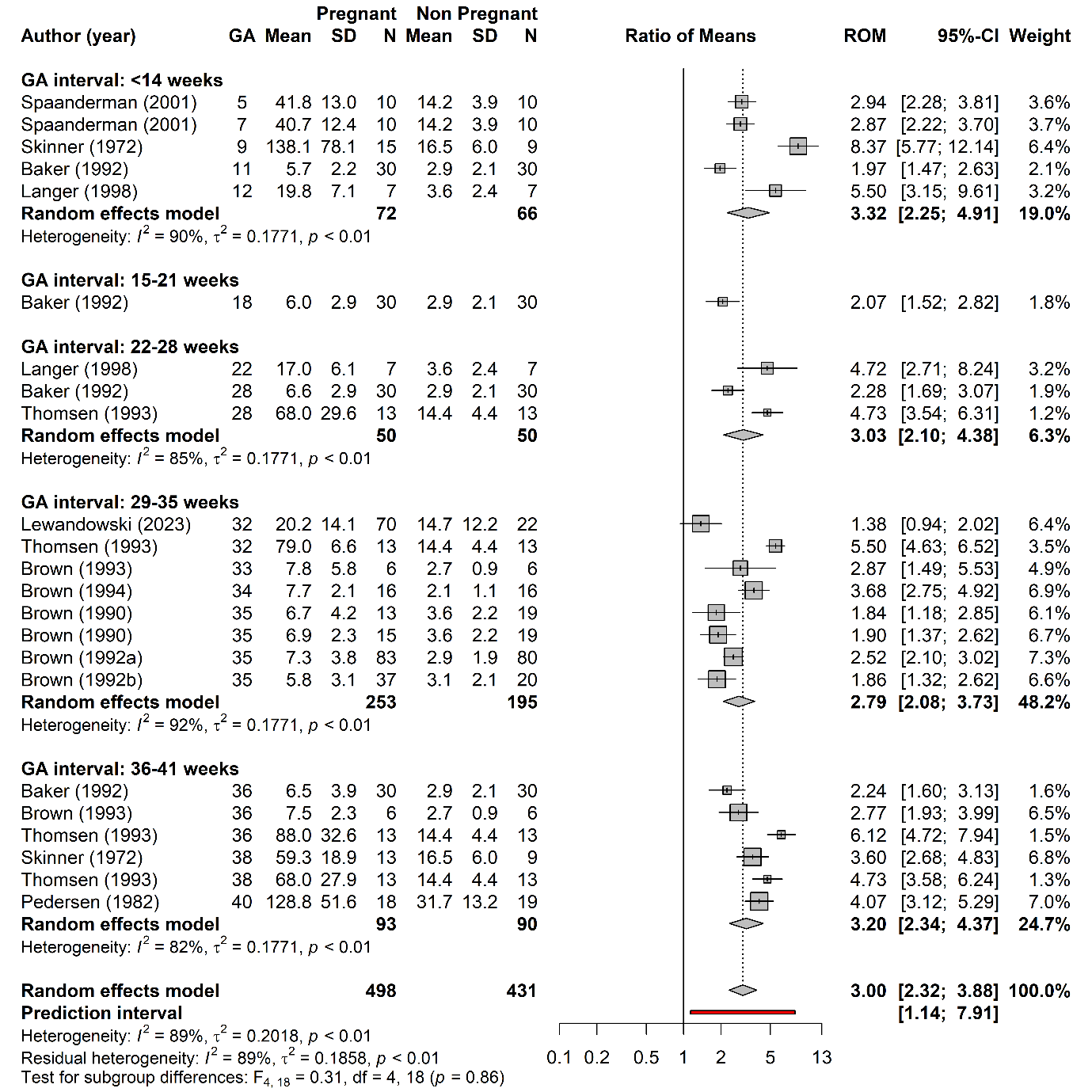


Figure S4.7. Forest plot of the ratio of means (ROM) of the active plasma renin concentrations (APRC) during healthy pregnancy at <14 weeks, 15-21 weeks, 22-28 weeks, 29-35 weeks and 36-41 weeks of gestation compared to reference values in non-pregnancy, preconception or postpartum. The analysis is restricted to studies that collected blood samples to determine renin concentrations in the morning. Studies that are reported more than once provide data for different gestational weeks within the same study. Only the first author of each study is given. *GA = gestational age in weeks, SD = standard deviation, CI = confidence interval.*

## Figure S4.8


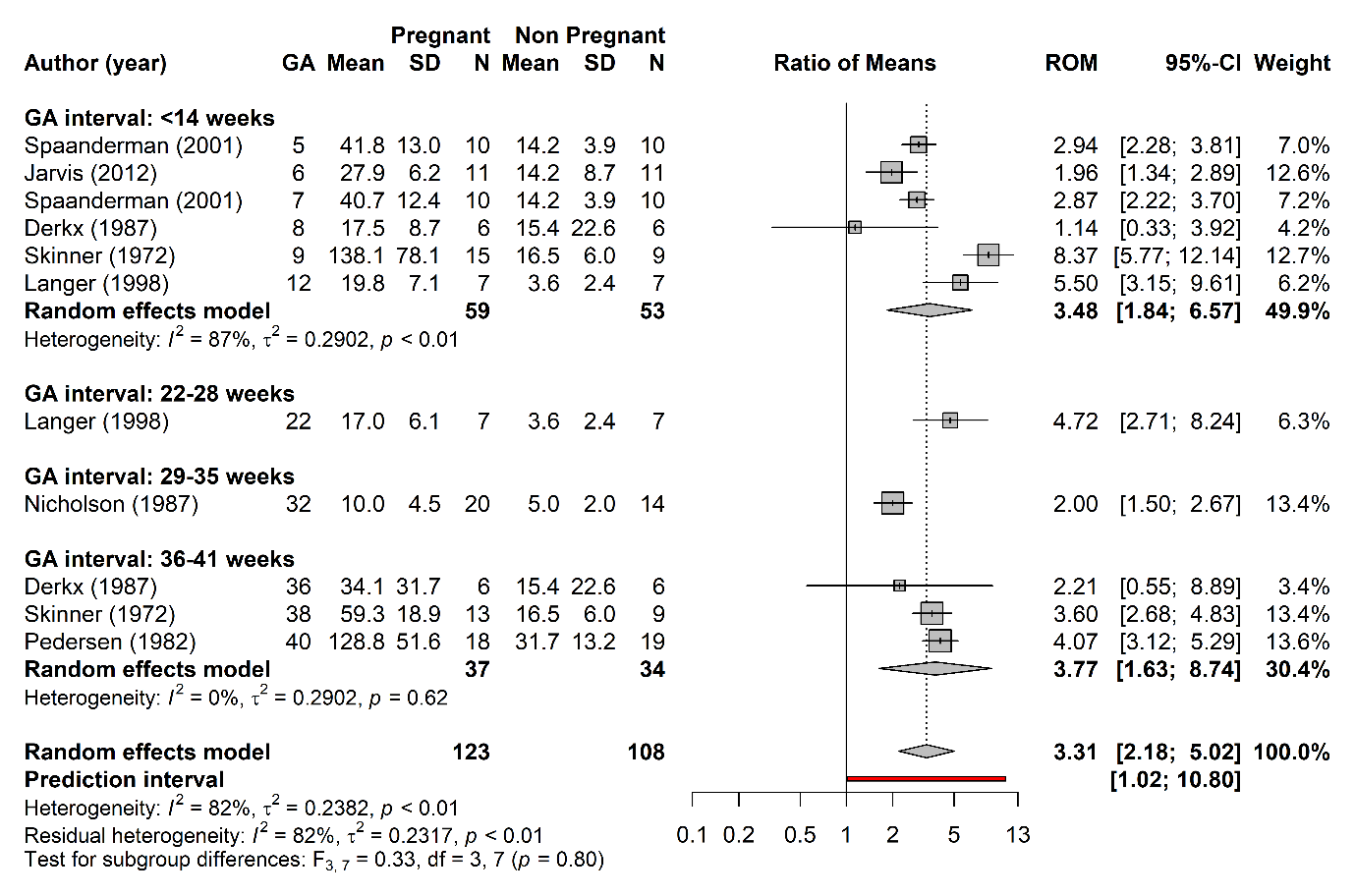


Figure S4.8. Forest plot of the ratio of means (ROM) of the active plasma renin concentrations (APRC) during healthy pregnancy at <14 weeks, 15-21 weeks, 22-28 weeks, 29-35 weeks and 36-41 weeks of gestation compared to reference values in non-pregnancy, preconception or postpartum. The analysis is restricted to studies that presented renin concentrations in mean and standard deviation. Studies that are reported more than once provide data for different gestational weeks within the same study. Only the first author of each study is given. *GA = gestational age in weeks, SD = standard deviation, CI = confidence interval.*

## Figure S4.9


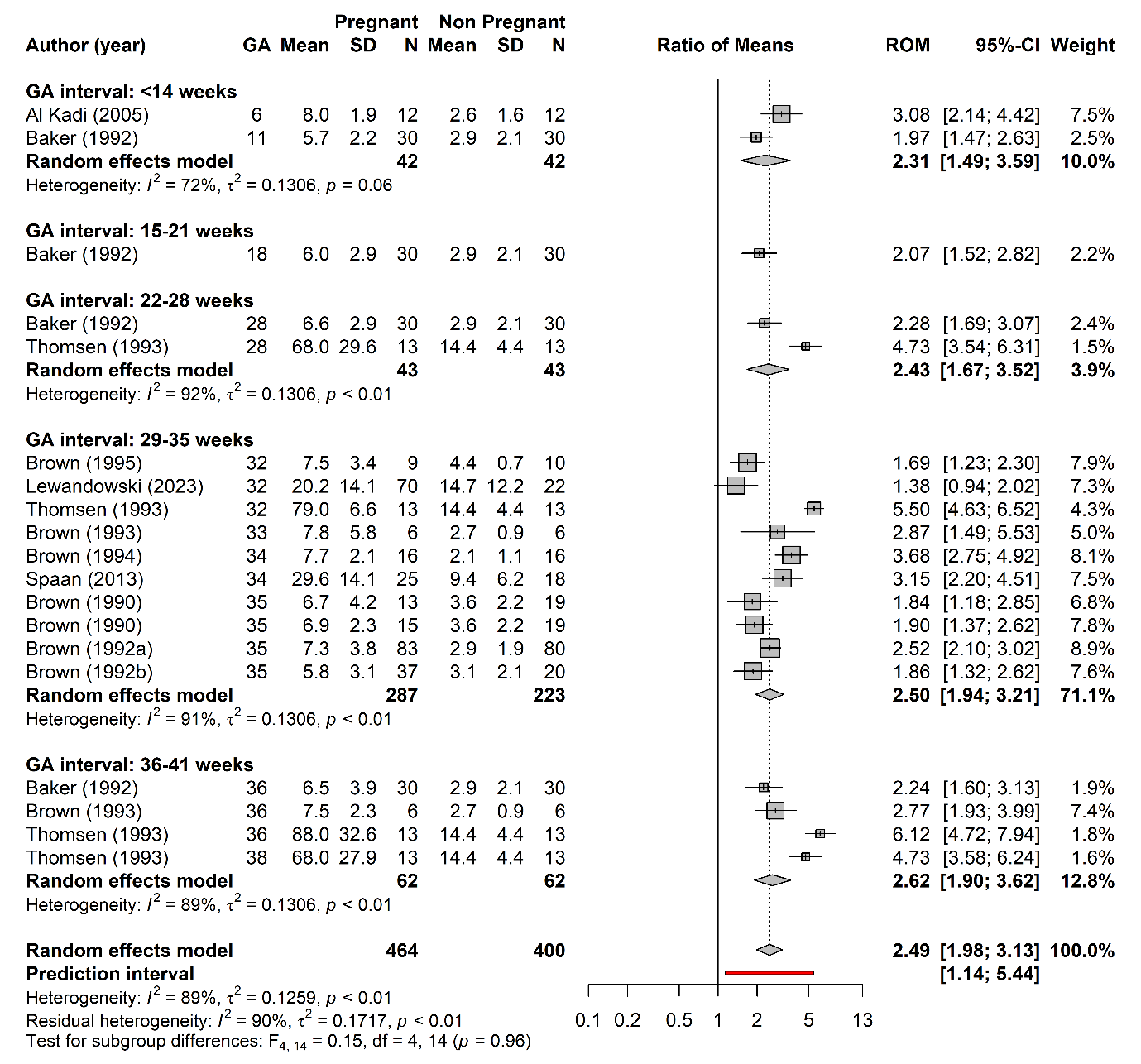


Figure S4.9. Forest plot of the ratio of means (ROM) of the active plasma renin concentrations (APRC) during healthy pregnancy at <14 weeks, 15-21 weeks, 22-28 weeks, 29-35 weeks and 36-41 weeks of gestation compared to reference values in non-pregnancy, preconception or postpartum. The analysis is restricted to studies that presented renin concentrations in median and interquartile range (IQR). Median and IQR are converted to mean and standard deviation for analysis. Studies that are reported more than once provide data for different gestational weeks within the same study. Only the first author of each study is given. *GA = gestational age in weeks, SD = standard deviation, CI = confidence interval.*

## Figure S4.10


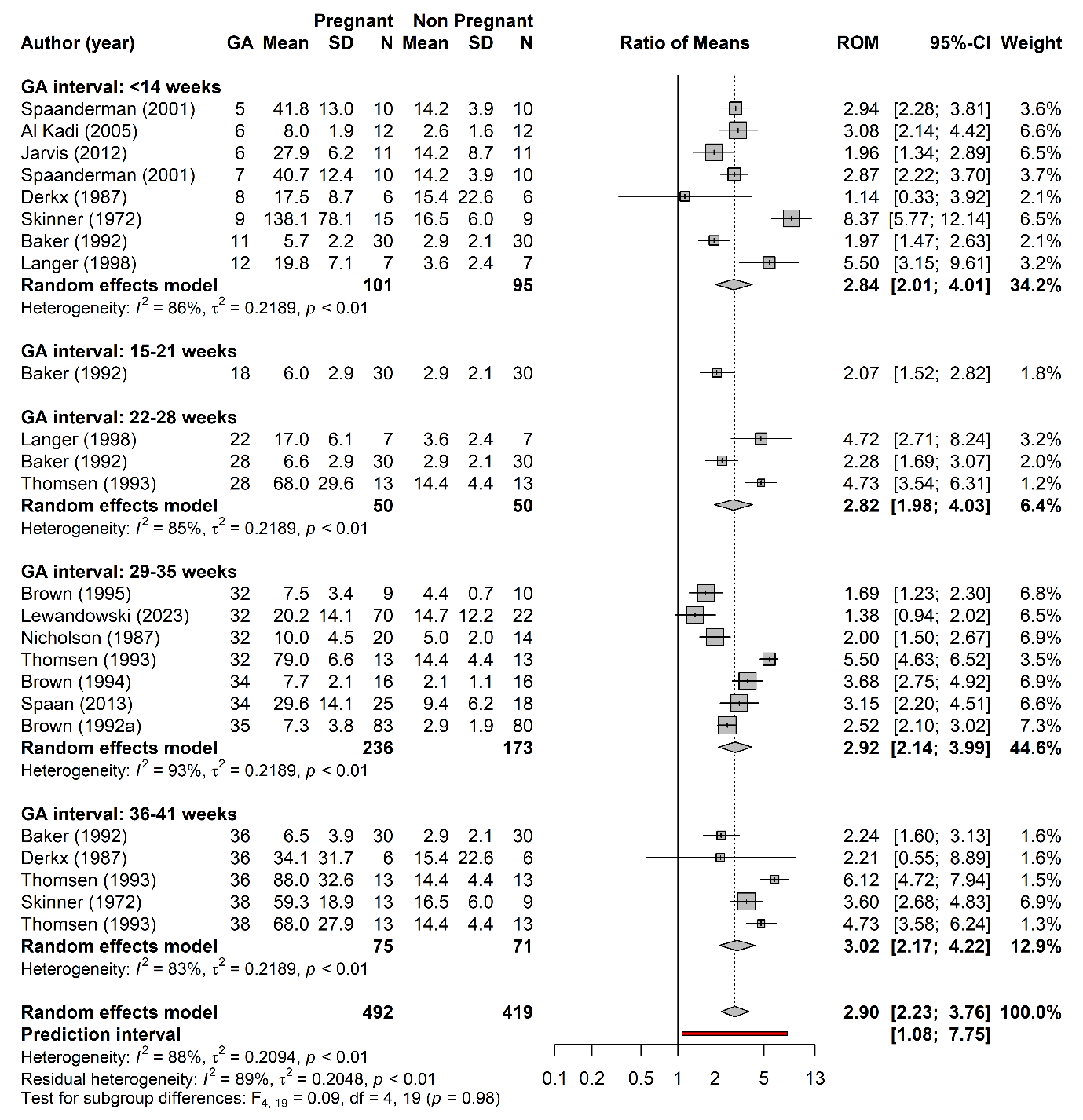


Figure S4.10. Forest plot of the ratio of means (ROM) of the active plasma renin concentrations (APRC) during healthy pregnancy at <14 weeks, 15-21 weeks, 22-28 weeks, 29-35 weeks and 36-41 weeks of gestation compared to reference values in non-pregnancy, preconception or postpartum. Studies that presented renin concentrations only in graphs are excluded in this analysis. Median and IQR are converted to mean and standard deviation for analysis. Studies that are reported more than once provide data for different gestational weeks within the same study. Only the first author of each study is given. *GA = gestational age in weeks, SD = standard deviation, CI = confidence interval.*

## Figure S4.11


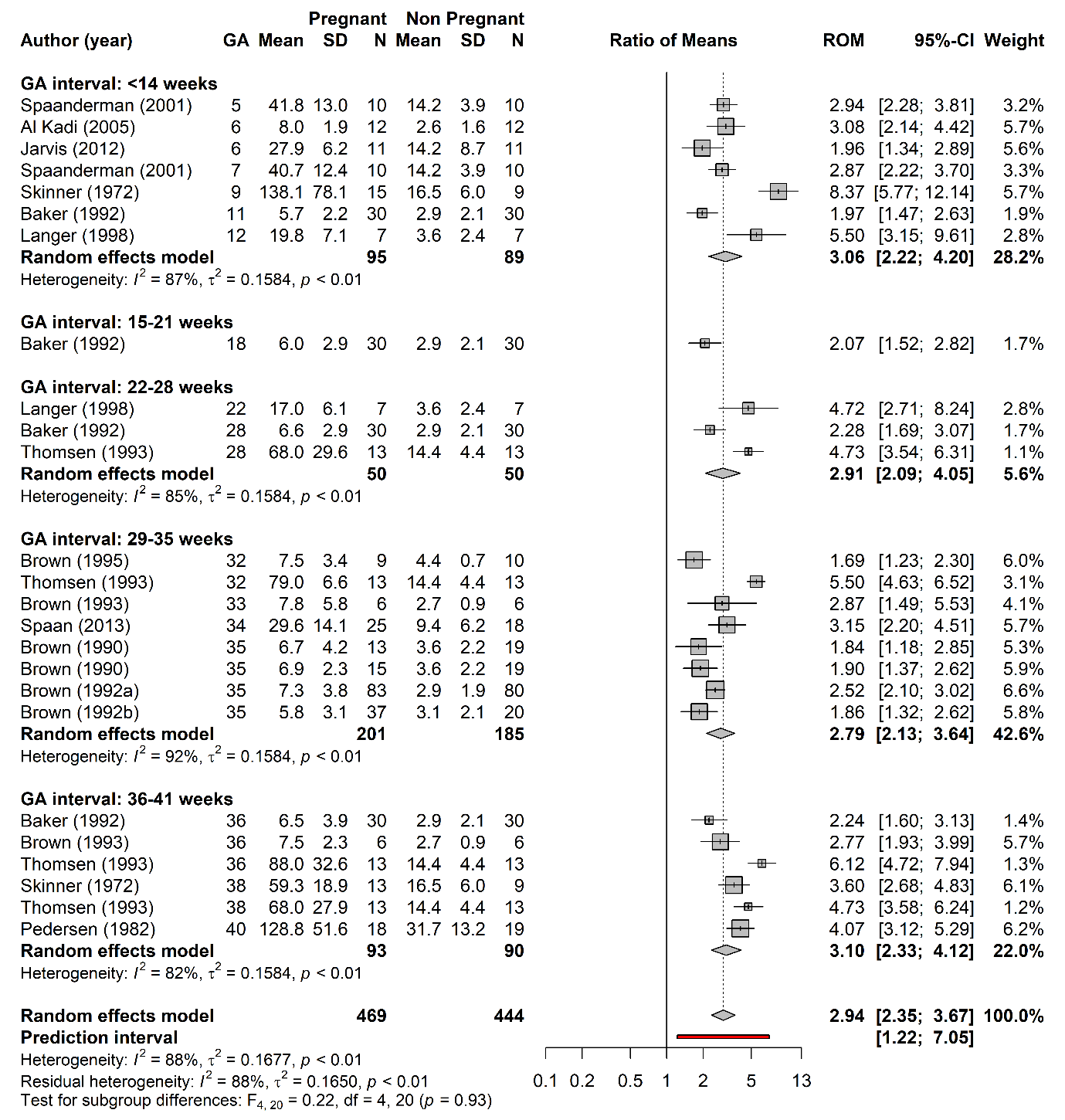


Figure S4.11. Forest plot of the ratio of means (ROM) of the active plasma renin concentrations (APRC) during healthy pregnancy at <14 weeks, 15-21 weeks, 22-28 weeks, 29-35 weeks and 36-41 weeks of gestation compared to reference values in non-pregnancy, preconception or postpartum. The analysis is restricted to studies that were classified as medium or high quality. Studies that are reported more than once provide data for different gestational weeks within the same study. Only the first author of each study is given. *GA = gestational age in weeks, SD = standard deviation, CI = confidence interval.*

## Figure S4.12


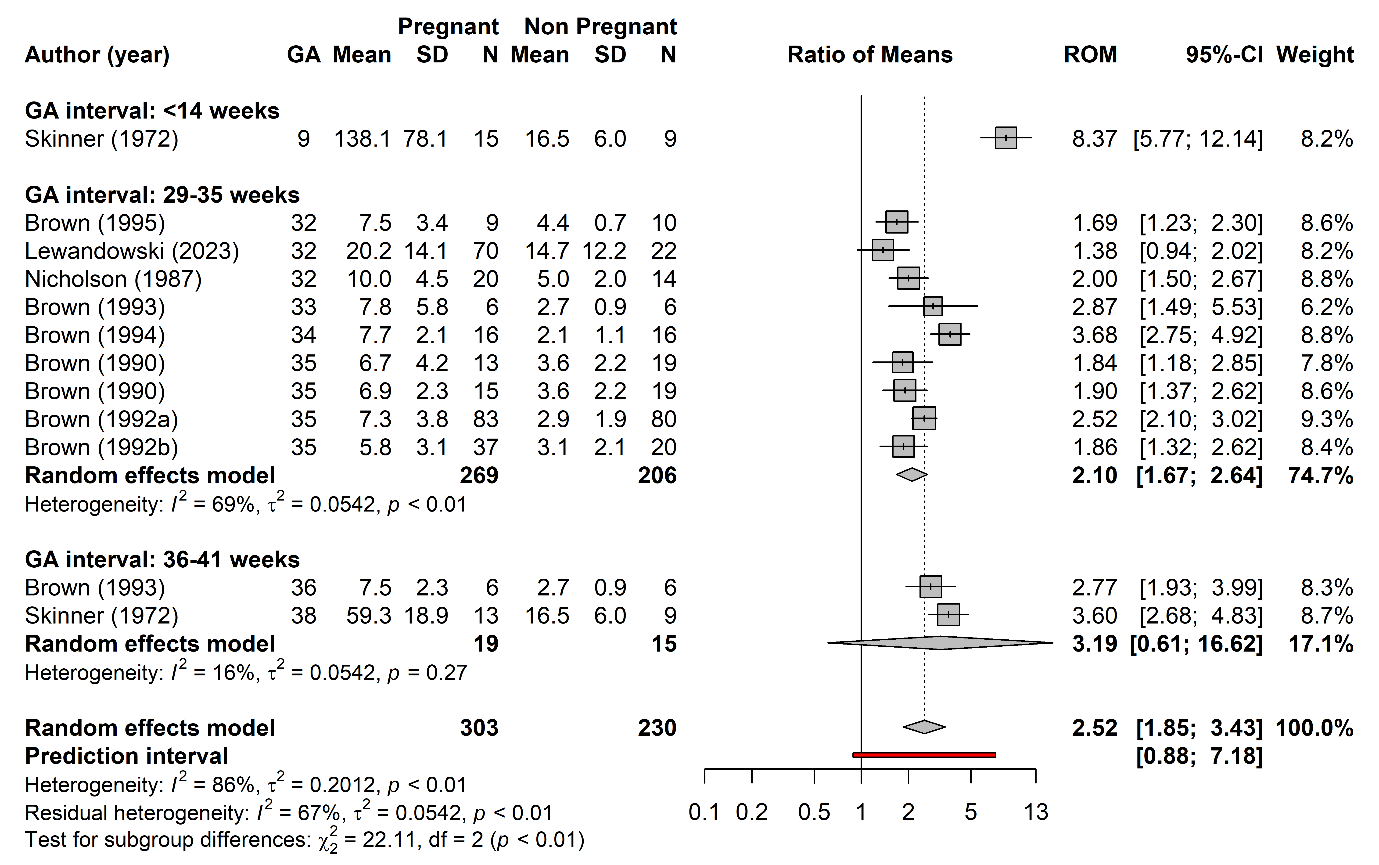


Figure S4.12. Forest plot of the ratio of means (ROM) of the active plasma renin concentrations (APRC) during healthy pregnancy at <14 weeks, 15-21 weeks, 22-28 weeks, 29-35 weeks and 36-41 weeks of gestation compared to reference values prior to pregnancy. The analysis is restricted to studies that measured APRC in the reference group prior to pregnancy or in a nonpregnant group. Studies that are reported more than once provide data for different gestational weeks within the same study. Only the first author of each study is given. *GA = gestational age in weeks, SD = standard deviation, CI = confidence interval.*

## Figure S4.13


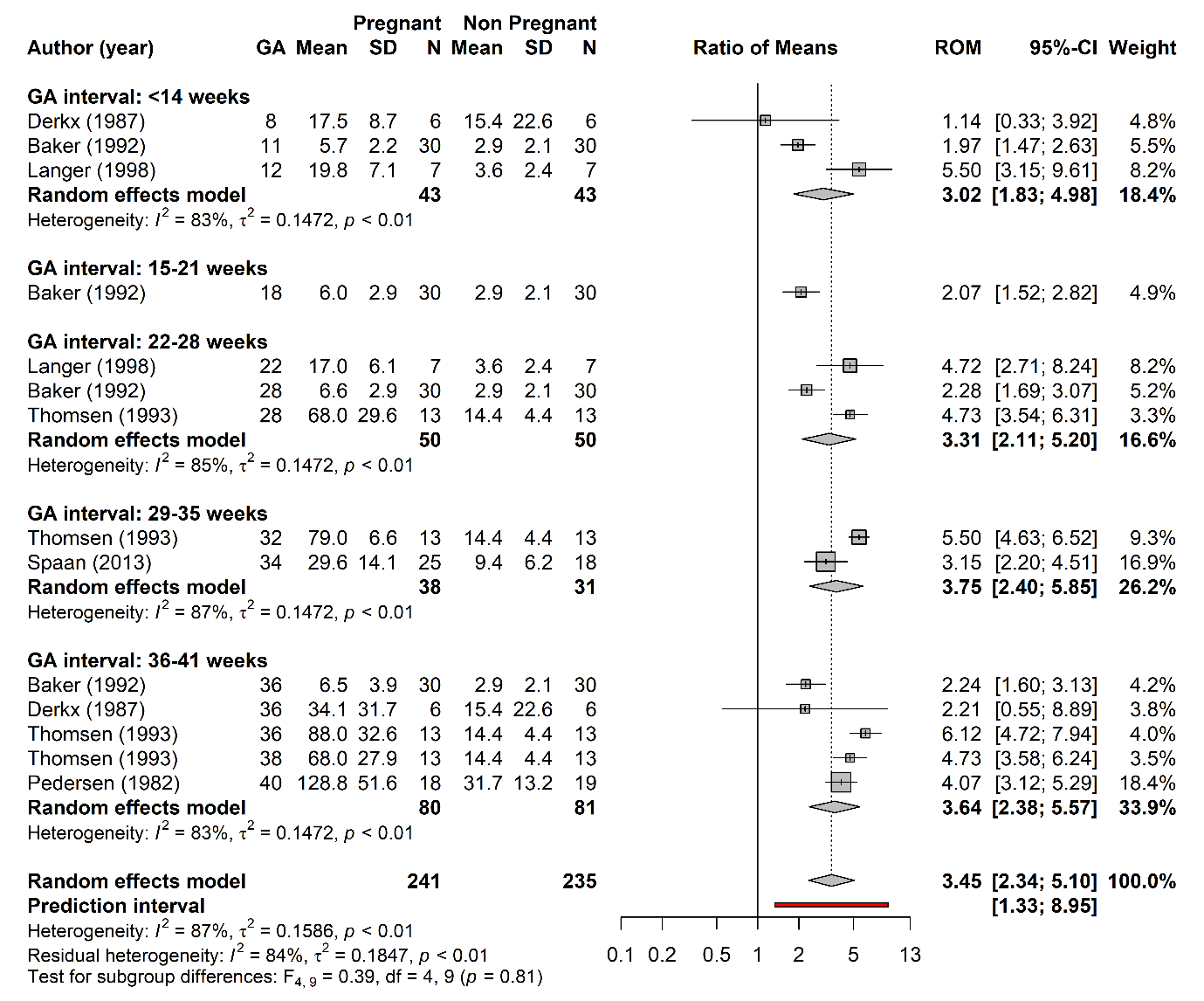


Figure S4.13. Forest plot of the ratio of means (ROM) of the active plasma renin concentrations (APRC) during healthy pregnancy at <14 weeks, 15-21 weeks, 22-28 weeks, 29-35 weeks and 36-41 weeks of gestation compared to reference values postpartum. The analysis is restricted to studies that measured APRC in the reference group postpartum. Studies that are reported more than once provide data for different gestational weeks within the same study. Only the first author of each study is given. *GA = gestational age in weeks, SD = standard deviation, CI = confidence interval.*

# Appendix S5: Sensitivity analyses of APRC in complicated pregnancy

## Figure S5.1


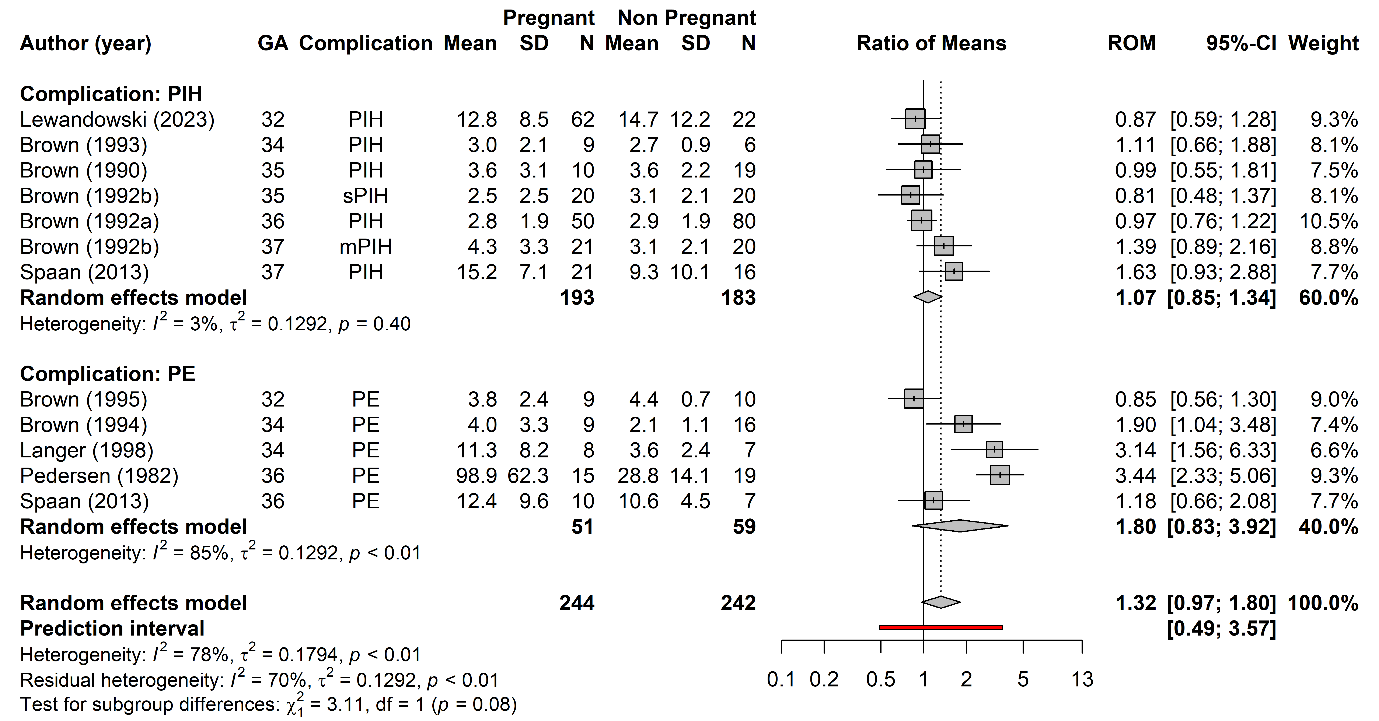


Figure S5.1. Forest plot of the ratio of means (ROM) of the active plasma renin concentrations (APRC) during hypertensive complicated pregnancy compared to reference values in non-pregnancy, preconception or postpartum. Studies are grouped by complication: pregnancy induced hypertension (PIH) or preeclampsia (PE). Studies that are reported more than once provide data for different gestational weeks within the same study. Only the first author of each study is given. *GA = gestational age in weeks, SD = standard deviation, CI = confidence interval.*

## Figure S5.2


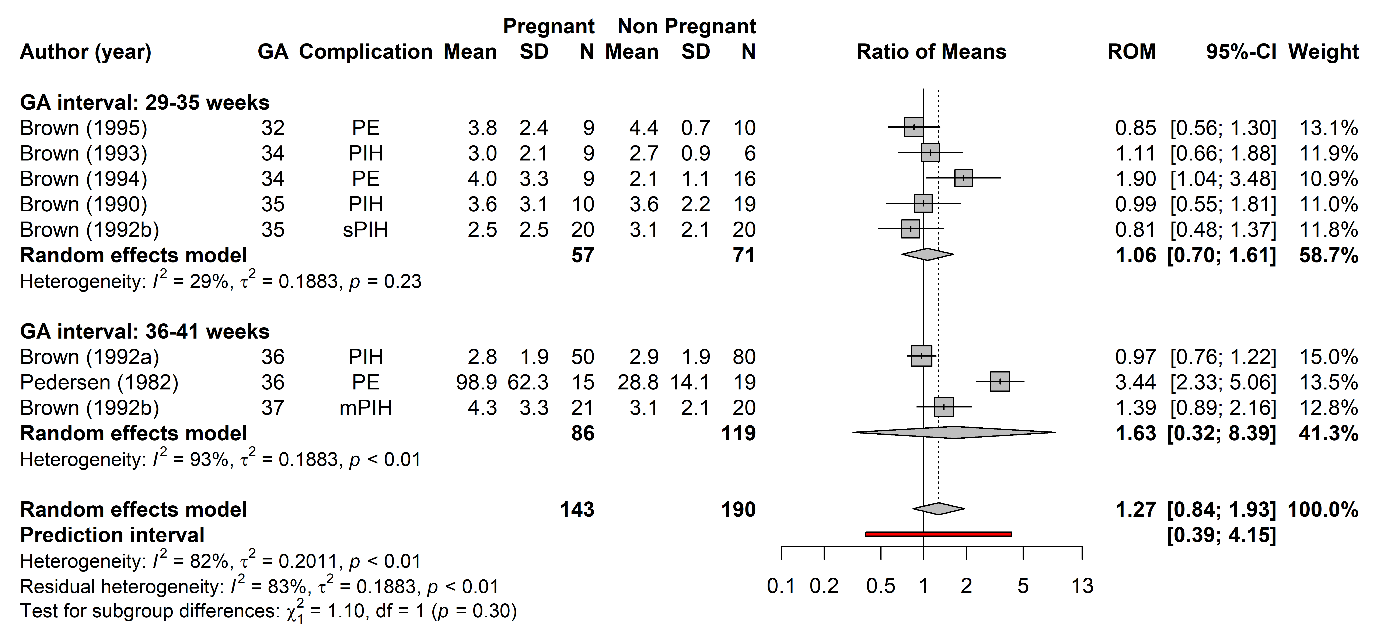


Figure S5.2. Forest plot of the ratio of means (ROM) of the active plasma renin concentrations (APRC) restricted to studies that used an activity assay during hypertensive complicated pregnancy at 29-35 weeks and 36-41 weeks of gestation compared to reference values in non-pregnancy, preconception or postpartum. Studies that are reported more than once provide data for different gestational weeks within the same study. Only the first author of each study is given. *GA = gestational age in weeks, SD = standard deviation, CI = confidence interval, PE = preeclampsia, PIH = pregnancy induced hypertension.*

## Figure S5.3


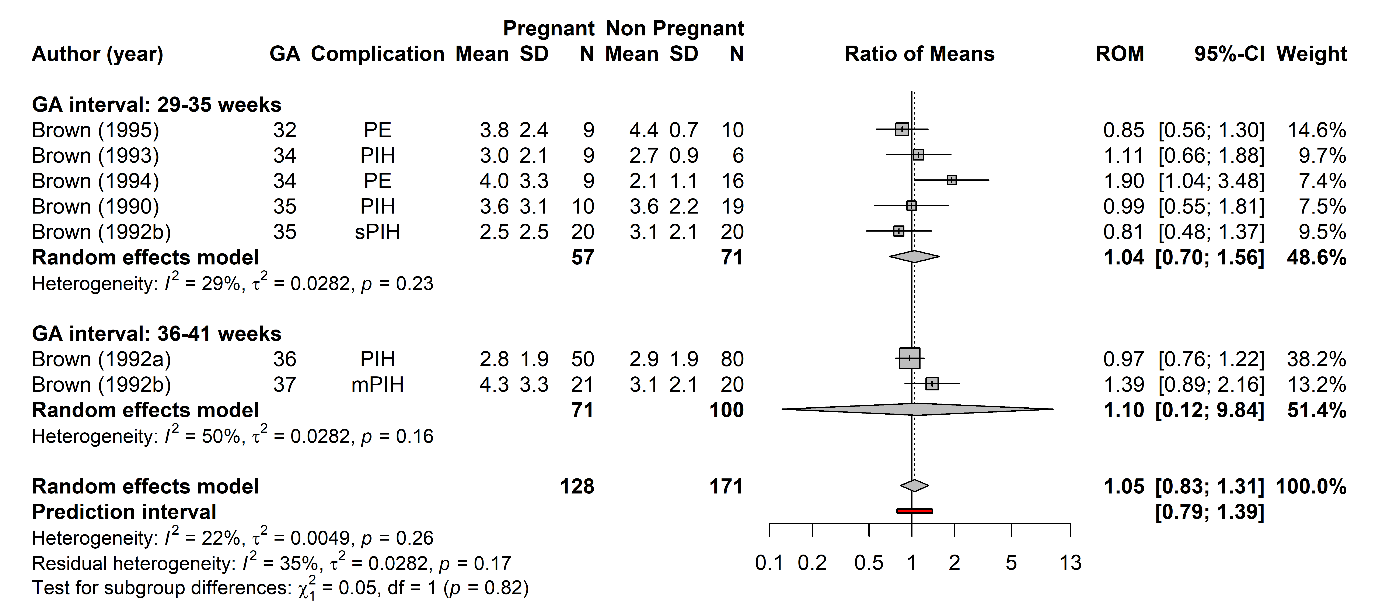


Figure S5.3. Forest plot of the ratio of means (ROM) of the active plasma renin concentrations (APRC) during hypertensive complicated pregnancy at 29-35 weeks and 36-41 weeks of gestation compared to reference values in non-pregnancy, preconception or postpartum. The analysis is restricted to studies that collected blood samples to determine renin concentrations in left lateral recumbency position. Studies that are reported more than once provide data for different gestational weeks within the same study. Only the first author of each study is given. *GA = gestational age in weeks, SD = standard deviation, CI = confidence interval, PE = preeclampsia, PIH = pregnancy induced hypertension.*

## Figure S5.4


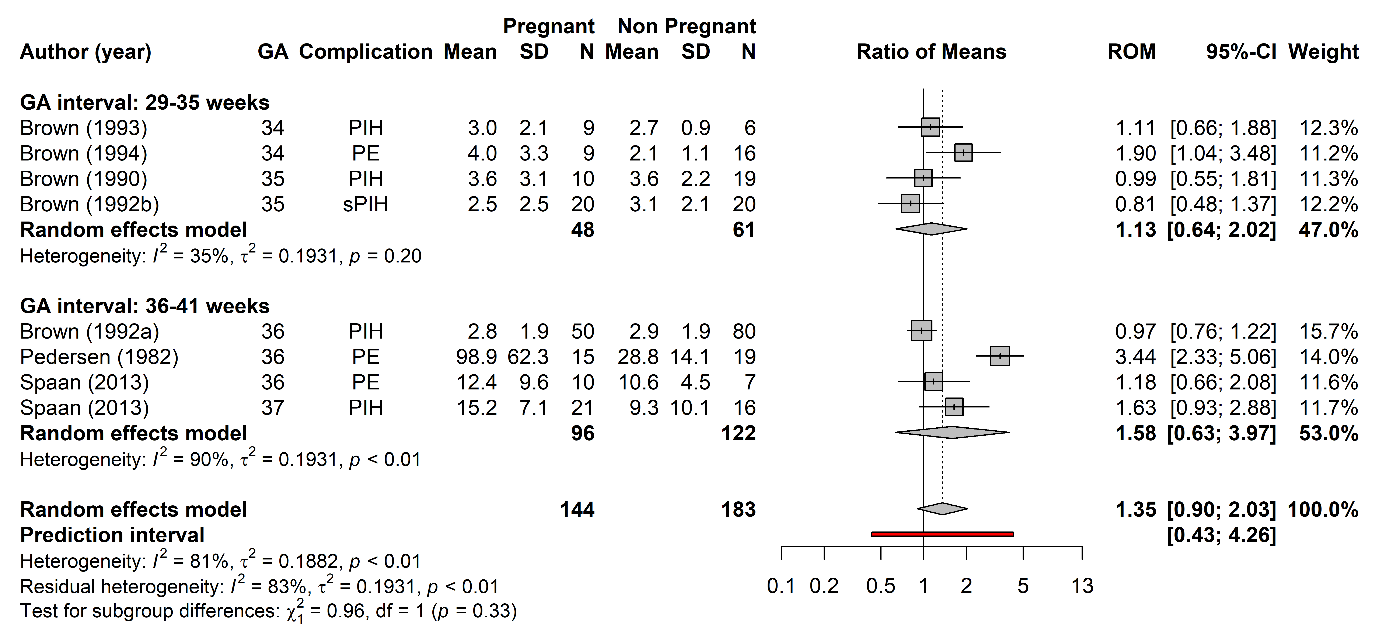


Figure S5.4. Forest plot of the ratio of means (ROM) of the active plasma renin concentrations (APRC) during hypertensive complicated pregnancy at 29-35 weeks and 36-41 weeks of gestation compared to reference values in non-pregnancy, preconception or postpartum. The analysis is restricted to studies that were classified as medium or high quality. Studies that are reported more than once provide data for different gestational weeks within the same study. Only the first author of each study is given. *GA = gestational age in weeks, SD = standard deviation, CI = confidence interval, PE = preeclampsia, PIH = pregnancy induced hypertension.*

## Figure S5.5


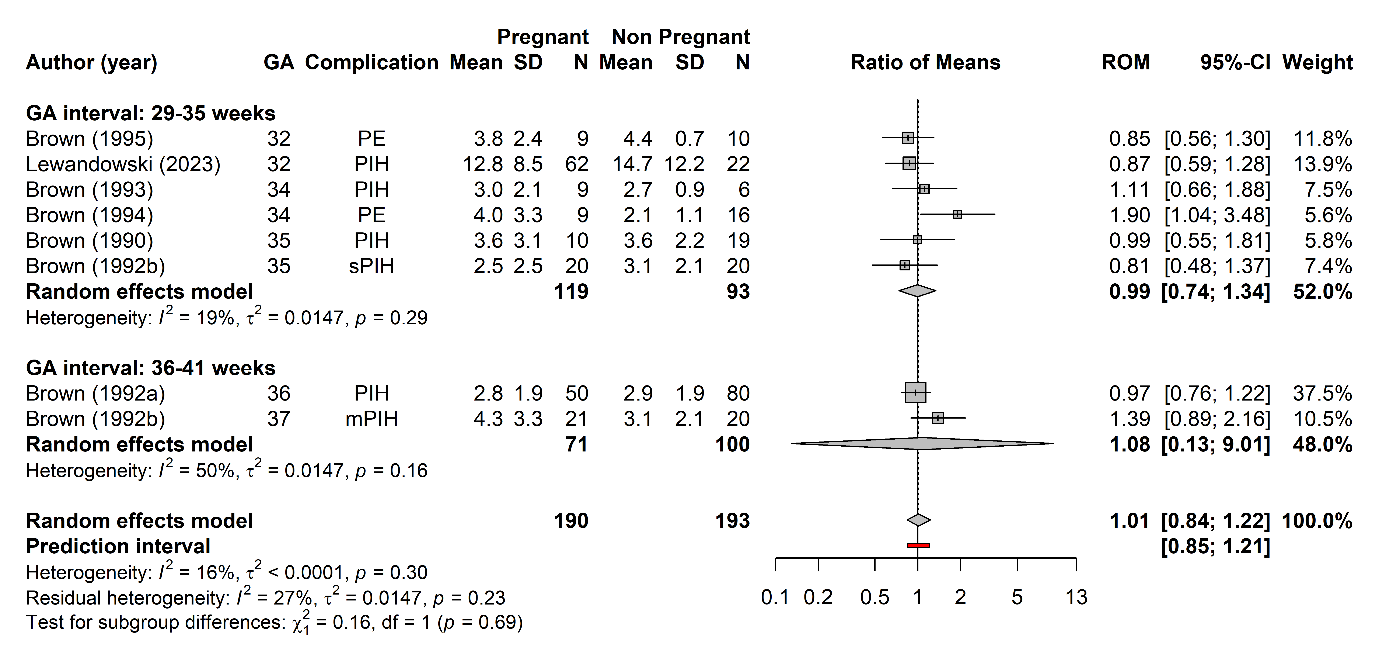


Figure S5.5. Forest plot of the ratio of means (ROM) of the active plasma renin concentrations (APRC) during hypertensive complicated pregnancy at 29-35 weeks and 36-41 weeks of gestation compared to reference values prior to pregnancy or in a non-pregnant control group. Studies that are reported more than once provide data for different gestational weeks within the same study. Only the first author of each study is given. *GA = gestational age in weeks, SD = standard deviation, CI = confidence interval, PE = preeclampsia, PIH = pregnancy induced hypertension.*
